# Neuronal C/EBPβ Shortens the Lifespan via Inactivating NAMPT

**DOI:** 10.1002/advs.202414871

**Published:** 2025-04-30

**Authors:** Bowei Li, Zhongyun Xie, Mengmeng Wang, Shuke Nie, Zhengjiang Qian, Xin Meng, Xia Liu, Seong Su Kang, Keqiang Ye

**Affiliations:** ^1^ Brain Cognition and Brain Disease Institute (BCBDI) Shenzhen Institutes of Advanced Technology (SIAT) Chinese Academy of Sciences Shenzhen Guangdong 518055 China; ^2^ University of Chinese Academy of Sciences Beijing 100049 China; ^3^ Department of Neurology Renmin Hospital of Wuhan University Wuhan Hubei 430060 China; ^4^ Department of Pathology and Laboratory Medicine Emory University School of Medicine Atlanta GA 30322 USA; ^5^ Faculty of Life and Health Sciences Shenzhen University of Advanced Technology (SUAT) Shenzhen Guangdong 518055 China

**Keywords:** asparagine endopeptidase (AEP), brain aging, CCAAT/enhancer binding protein β (C/EBPβ), nicotinamide adenine dinucleotide (NAD^+^), nicotinamide phosphoribosyltransferase (NAMPT)

## Abstract

The brain plays a central role in aging and longevity in diverse model organisms. Morphological and functional alteration in the aging brain elicits age‐associated neuronal dysfunctions. However, the primary mechanism deteriorating the brain functions to regulate the aging process remains incompletely understood. Here, it is shown that neuronal CCAAT/enhancer binding protein β (C/EBPβ) escalation during aging dictates the frailty and lifespan via inactivating nicotinamide phosphoribosyltransferase (NAMPT). Upregulated C/EBPβ drives neuronal senescence and neuronal loss, associated with NAMPT fragmentation by active asparagine endopeptidase (AEP), leading to nicotinamide adenine dinucleotide (NAD^+^) depletion. Knockout of AEP or expression of AEP‐resistant NAMPT N136A mutant significantly elongates the lifespan of neuronal‐specific Thy 1‐C/EBPβ transgenic mice. Overexpression of the *C. elegans* C/EBPβ ortholog *cebp‐2* in neurons shortens lifespan and decreases NAD^+^ levels, which are restored by feeding nicotinamide mononucleotide (NMN) or AEP inhibitor #11a. Feeding NMN or #11a substantially ameliorates the cognitive and motor impairments of Thy 1‐C/EBPβ mice and increases the life expectancy. Notably, #11a demonstrates a better therapeutic effect than NMN in improving aging phenotype in Thy 1‐C/EBPβ transgenic mice, which show accelerated aging features. Hence, blockade of AEP via therapeutic intervention may provide an unprecedented strategy for fighting aging and various age‐associated diseases.

## Graphical abstract

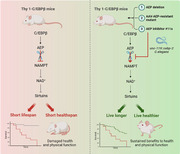

## 1 Introduction

Brain functions decline with age and cause significant impairment in quality of life owing to social, cognitive, and physical disability. Age‐associated neuronal dysfunction is attributable to morphological and functional variation in the brain, accompanied by ultrastructural changes in neurons and glia.^[^ ^1^ ^]^ Brain atrophy takes place especially in the prefrontal cortex and the hippocampus during aging.^[^ ^2^ ^]^ The aged brain displays chronic low‐grade neuroinflammation and increased microglia reactivity. Activated microglia produce pro‐inflammatory cytokines, with additional inflammatory cytokines from the peripheral immune system that penetrate the brain because of age‐increased blood–brain barrier permeability. Elevated levels of interleukin‐1β (IL‐1β), interleukin‐6 (IL‐6), and tumour necrosis factor‐α (TNF‐α) in the aged brain are associated with reduced levels of anti‐inflammatory cytokines such as IL‐10 and IL‐4.^[^ ^1^ ^]^

CCAAT/enhancer binding proteins (C/EBPs) belong to the basic‐leucine zipper DNA‐binding protein family and are implicated in CNS inflammation.^[^ ^3^ ^]^ C/EBPs regulate the expression of genes critical to glia activation, and C/EBP binding sites are located in the promoter regions of numerous cytokines and other pro‐inflammatory genes.^[^ ^4^ ^]^ In addition, C/EBPs also play important roles in transcription underlying more complex brain functions, such as learning and memory.^[^ ^5^ ^]^ Notably, C/EBPβ is age‐dependently escalated in neurons, and its deficiency provides cerebral protection following excitotoxic injury.^[^ ^6^ ^]^ Moreover, C/EBPβ is upregulated in Alzheimer's disease (AD) brains.^[^ ^7^ ^]^ We have shown that it temporally regulates asparagine endopeptidase (AEP, also called LGMN or δ‐secretase) expression, which cleaves both APP N585 and Tau N368, triggering AD pathogenesis.^[^ ^8^, ^9^, ^10^, ^11^, ^12^ ^]^ Neuronal C/EBPβ/AEP pathway drives GABAergic neuronal loss and modulates neural excitation and lifespan via repressing REST/forkhead box protein O (FOXO).^[^ ^13^ ^]^ C/EBPβ mRNA contains three alternative translational isoforms (LAP, LAP* and LIP). Interestingly, female C/EBPβ LAP (transcription activator) only mice show increased median life span by 20%.^[^ ^14^ ^]^ By contrast, mice only expressing the LIP but lacking LAP isoform display reduced lifespan.^[^ ^15^ ^]^ Hence, C/EBPβ is implicated in the regulation of longevity.

Nicotinamide adenine dinucleotide (NAD^+^) is an enzyme cofactor or co‐substrate in many essential biological pathways. NAD^+^ can be synthesized from several different precursors, among which nicotinamide is the substrate predominantly used in mammals. NAD^+^ augmentation, via its precursors nicotinamide riboside (NR) and nicotinamide mononucleotide (NMN), alleviates cognitive impairment, Aβ, and Tau pathologies in different AD animal models and AD iPSC‐derived neurons.^[^ ^16^, ^17^, ^18^ ^]^ Interestingly, treatment of AD mice with NR reduced neuroinflammation, attenuated DNA damage, and prevented cellular senescence, in part, through a cyclic GMP‐AMP synthase–stimulator of interferon genes‐dependent pathway.^[^ ^19^ ^]^ NAD^+^ augmentation also rescues the mitochondrial and aging phenotype in xeroderma pigmentosum group A (XPA) cells and *xpa‐1* worms.^[^ ^20^ ^]^ Nicotinamide phosphoribosyltransferase (NAMPT) is the rate‐limiting enzyme that catalyzes NAD^+^ biosynthesis from nicotinamide.^[^ ^21^ ^]^ Forebrain excitatory neurons mainly use intracellular NAMPT‐mediated NAD^+^ biosynthesis to mediate neuronal survival and functions.^[^ ^22^ ^]^ Mice lacking NAMPT in forebrain excitatory neurons (CaMKIIα‐NAMPT^−/−^) show hippocampal and cortical atrophy, astrogliosis, microgliosis, and abnormal CA1 dendritic morphology, demonstrating impaired motor and cognitive behaviors.^[^ ^22^ ^]^ NAMPT knockdown in the CA1 region recapitulates hippocampal cognitive phenotypes in old mice, and NMN administration improves cognitive functions.^[^ ^23^ ^]^ Depletion of NAMPT disturbs mitochondrial homeostasis and causes neuronal degeneration in the hippocampus.^[^ ^24^ ^]^ Sirtuins are an evolutionally conserved family of NAD^+^‐dependent deactylases and play a critical part in aging and longevity control in diverse model organisms, including yeast, worms, flies, and mice.^[^ ^25^, ^26^ ^]^ Hence, advances in understanding the molecular and cellular mechanisms underlying NAMPT/NAD^+^/Sirtuin 1 (SIRT1)‐dependent neuronal resilience will provide novel approaches for promoting healthy brain aging and treating a range of neurological diseases.^[^ ^27^ ^]^

In the current study, we show that neuronal C/EBPβ elicits neuronal senescence and triggers cognitive deficits and motor impairments via inactivating NAMPT, which is cleaved by active AEP, shortening the lifespan. Knockout of AEP or blockade of NAMPT cleavage attenuates these defects in neuronal Thy 1‐C/EBPβ transgenic (Tg) mice. Administration of NMN or AEP‐specific inhibitor #11a ameliorates these events and elongates life expectancy in both Thy 1‐C/EBPβ transgenic mice and worms, suggesting that blockade of AEP via therapeutic intervention may counteract aging and affect longevity.

## 2 Results

### 2.1 C/EBPβ/AEP Escalation in the Aging Brain

**Figure 1:**
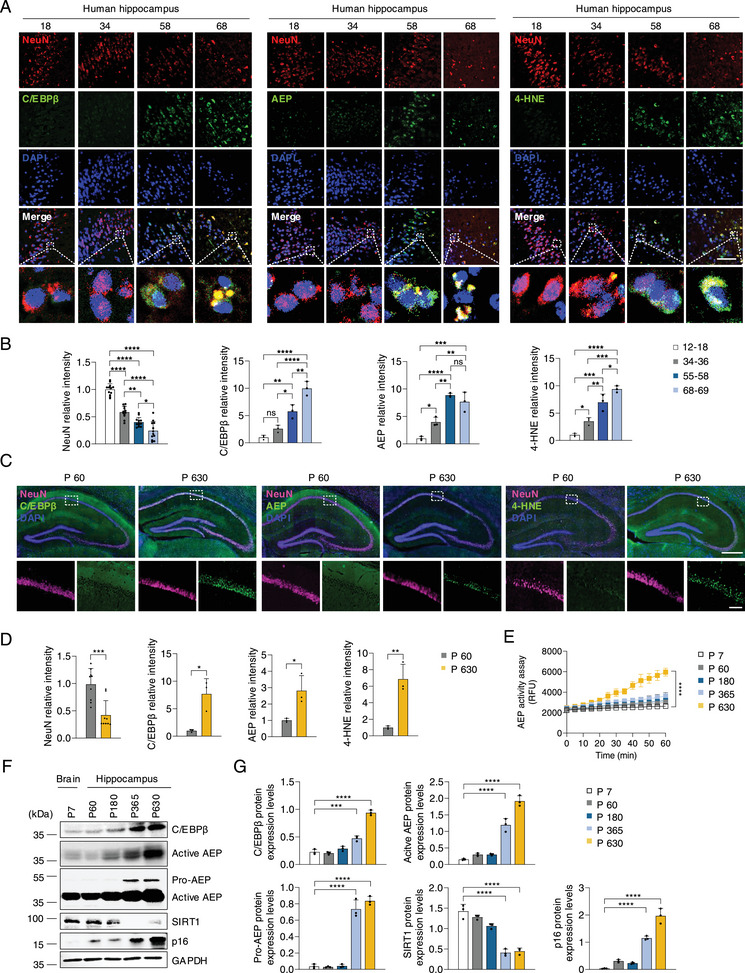
C/EBPβ/AEP escalation in the aging brain. A) Human hippocampus sections of different age (12–18, 34–36, 55–58, 68–69 years old) groups showing NeuN, C/EBPβ, AEP, and 4‐HNE protein expression (n = 3). Scale bars, 50 µm. B) Quantification of relative fluorescence intensity in A). C) IF staining of NeuN, C/EBPβ, AEP, and 4‐HNE in the brain from different ages (60, 630 days postpartum) of C57BL/6 mice (n = 3). Scale bars, 500 µm, top, 50 µm, bottom. D) Quantification of relative fluorescence intensity in C). E) AEP activity in the hippocampus from different ages (7, 60, 180, 365, 630 days postpartum) of C57BL/6 mice (n = 3). F) Western blot analysis of C/EBPβ, AEP, SIRT1, and p16 in the mouse brain or hippocampus during aging (n = 3). G) Quantification of protein levels in D). All data are presented as the mean ± SEM from 3 to 6 independent experiments. *, P < 0.05; **, P < 0.01; ***, P < 0.001; ****, P < 0.0001.

First, we assessed expression of Cebpb and Lgmn using the publicly available human RNA seq dataset (The Human Protein Atlas) and found that Cebpb and Lgmn were prominently expressed in neuronal cell populations in different regions of the brain (Figure S1A,B, Supporting Information). To investigate whether neuronal C/EBPβ and AEP are implicated in the brain aging process, we employed human and mouse brain samples for different time points. Immunofluorescent (IF) co‐staining using anti‐NeuN, a specific neuron marker, and C/EBPβ, AEP revealed that hippocampal neurons decreased in a time‐dependent manner. C/EBPβ and AEP were progressively escalated in hippocampal neurons, inversely correlated with the gradual reduction of neurons. Moreover, 4‐HNE, a biomarker for oxidative stress, gradually escalated in the hippocampus in the brains (**Figure** **1**A,B). In the aging mouse brain, IF co‐staining with NeuN and C/EBPβ, AEP, 4‐HNE on the hippocampus sections from mice of different ages also exhibited a similar pattern (Figure 1C,D). AEP enzymatic activities in the hippocampus area were increased in a time‐dependent manner (Figure 1E). Immunoblotting (IB) revealed that C/EBPβ was also enhanced with time, as was the downstream target AEP. The 37 kDa active form was tightly correlated with the upstream effector, accompanied by the decline of SIRT1 (the longevity protein) and the accumulation of p16 (the aging marker) (Figure 1F,G). Thus, neuronal C/EBPβ/AEP were gradually activated in the aging human and mouse brain.

### 2.2 C/EBPβ Overexpression Elicits Neuronal Senescence Via Activating AEP

**Figure 2:**
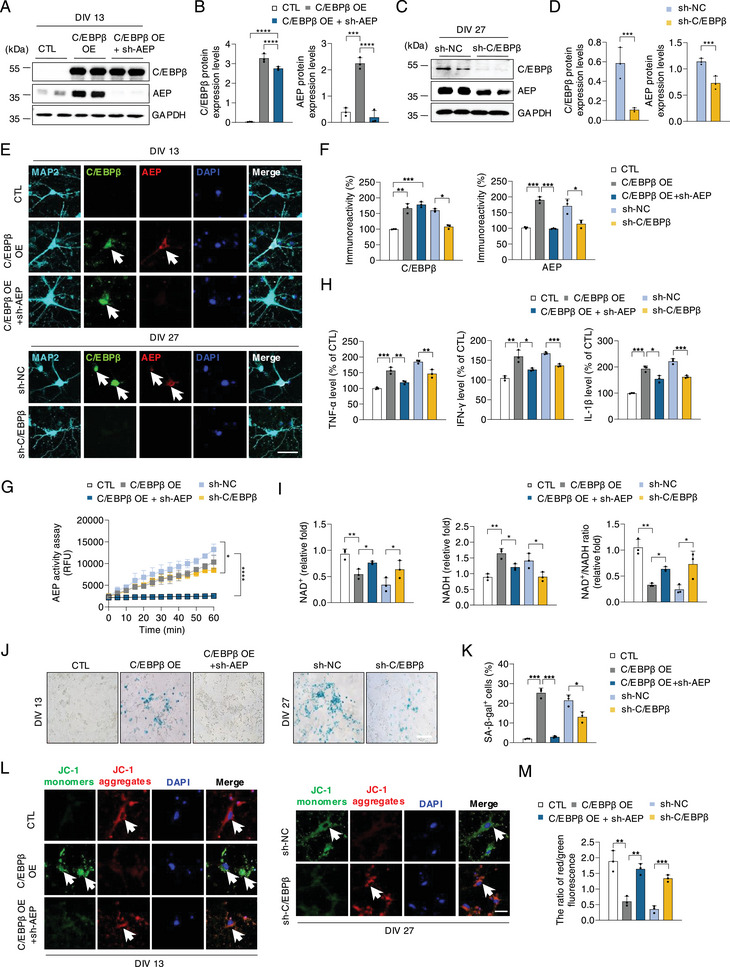
C/EBPβ overexpression elicits neuronal senescence via activating AEP. LV‐CTL, LV‐C/EBPβ, or LV‐C/EBPβ + LV‐sh‐AEP were infected primary neurons (DIV 13). LV‐sh‐NC or LV‐sh‐C/EBPβ were transfected into primary neurons (DIV 27). A) Western blot analysis of C/EBPβ, AEP protein levels in primary neurons (DIV 13) infected with LV‐CTL, LV‐C/EBPβ, or LV‐C/EBPβ + LV‐sh‐AEP. B) Quantification of protein levels in A). C) Western blot analysis of C/EBPβ, AEP protein levels in primary neurons (DIV 27) infected with LV‐sh‐NC or LV‐sh‐C/EBPβ. D) Quantification of protein levels in C). E) IF staining of MAP2, C/EBPβ, and AEP in the indicated group (n = 3). Scale bars, 50 µm. F) Quantification of relative fluorescence intensity in E). G) AEP activity in the indicated group (n = 3). H) qPCR analysis of senescence‐associated inflammatory cytokine genes in the indicated group (n = 3). I) NAD^+^ levels in the indicated group (n = 3). J) Representative images of senescence‐associated β‐galactosidase (SA‐β‐Gal) staining in the indicated group (n = 3). Scale bars, 100 µm. K) Quantification of SA‐β‐Gal^+^ cells in J). L) Representative images of the ratio of aggregates (red)/monomers (green) of JC‐1 staining in the indicated group (n = 3). Scale bars, 50 µm. M) Quantification of the ratio of red/green fluorescence in L). All data are presented as the mean ± SEM from 3 to 6 independent experiments. *, P < 0.05; **, P < 0.01; ***, P < 0.001; ****, P < 0.0001.

To assess whether C/EBPβ escalation drives neuronal senescence, we overexpressed C/EBPβ in young primary hippocampal neurons (DIV 13) with adeno‐associated virus (AAV) virus and found that AEP was greatly enhanced in comparison with the control virus (**Figure** **2**A,B). On the other hand, deletion of C/EBPβ from aging primary neurons (DIV 27) evidently reduced active AEP (Figure 2C,D). IF co‐staining with microtubule‐associated protein 2 (MAP2), a long, filamentous molecule thought to cross‐link the dendritic cytoskeleton, and C/EBPβ, AEP also exhibited a similar pattern (Figure 2E,F). AEP enzymatic activities were confirmed by a quantification assay (Figure 2G). Subsequently, we examined a variety of cell senescence markers. Since inflammation is involved in brain aging, we tested inflammatory cytokines transcription. Consequently, IL‐1β, TNFα, and INF‐γ levels oscillated with C/EBPβ expression patterns, and depletion of AEP partially reverses this effect (Figure 2H). NAD^+^ levels decline with aging and in some age‐related diseases, and a reduction in NAD^+^ affects all the hallmarks of aging.^[^ ^28^ ^]^ The NAD^+^ detection assay showed that NAD^+^ concentrations were reduced from DIV 13 to 27, which was further suppressed by C/EBPβ overexpression, and depletion of AEP significantly increased NAD^+^ levels. On the contrary, knocking down C/EBPβ substantially elevated NAD^+^ in DIV 27 neurons. NADH contents were conversely coupled with NAD^+^ (Figure 2I). Senescence test in DIV 13 neurons showed that C/EBPβ overexpression stimulated β‐gal staining, which was blocked when AEP was knocked down. As a result, β‐gal signals were highly elevated in DIV 27 neurons that were abrogated when C/EBPβ was deleted (Figure 2J,K). Mitochondrial dysfunction, operationally defined as a decreased mitochondrial membrane potential, are hallmarks of cell senescence.^[^ ^29^ ^]^ We monitored the mitochondrial membrane potential by JC‐1 dye. The results demonstrated that JC‐1 monomer, a biomarker for mitochondrial decreased membrane potential, aggregated in neurons with C/EBPβ overexpression, and depletion of AEP significantly converted JC‐1 from monomer to aggregate, indicating an increase in mitochondrial membrane potential (Figure 2L,M). Thus, C/EBPβ modulates neuronal senescence via activating AEP.

### 2.3 Knockout of AEP Ameliorates Age‐Related Impairments and Elongates the Short Lifespan Caused by Neuronal C/EBPβ Overexpression in Mice

**Figure 3:**
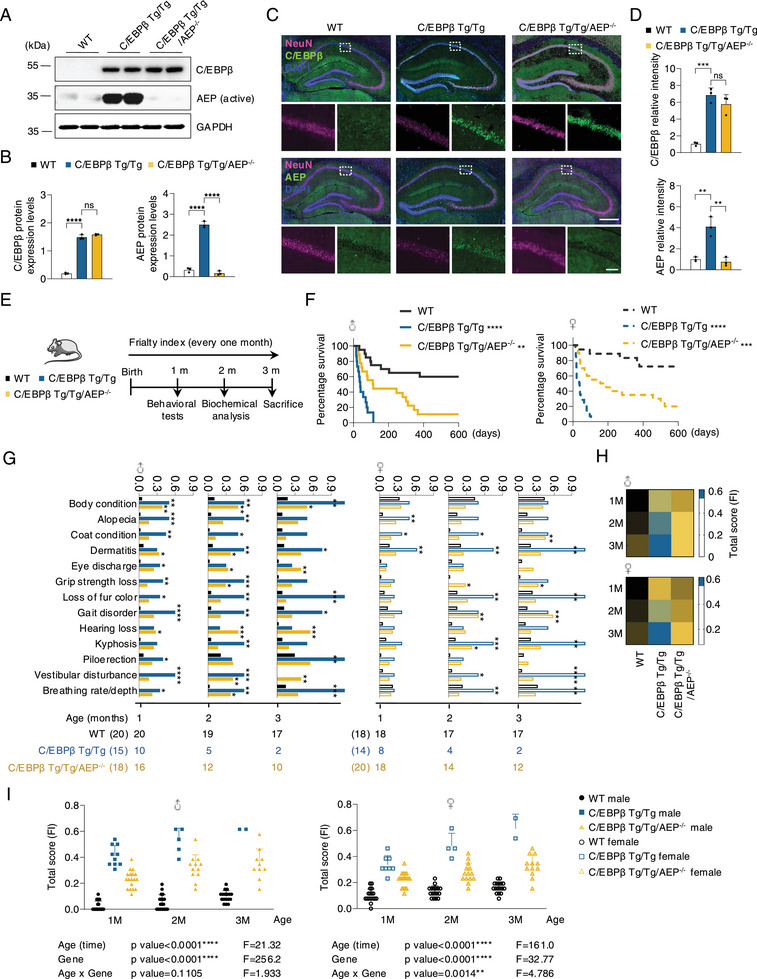
Knockout of AEP extends the short lifespan and alleviates age‐associated frailty caused by neuronal C/EBPβ overexpression in mice. A) Western blot analysis of C/EBPβ, AEP protein levels in wild‐type (WT), Thy 1‐C/EBPβ Tg/Tg, Thy 1‐C/EBPβ Tg/Tg/AEP^−/−^ mice (n = 3). B) Quantification of protein levels in A). C) IF staining of NeuN, C/EBPβ, and AEP in WT, Thy 1‐C/EBPβ Tg/Tg, Thy 1‐C/EBPβ Tg/Tg/AEP^−/−^ mice (n = 3). Scale bars, 500 µm, top, 50 µm, bottom. D) Quantification of relative fluorescence intensity in C). E) The timeline of behavioral tests, biochemical analysis, and frailty index (FI) monitor to WT, Thy 1‐C/EBPβ Tg/Tg, Thy 1‐C/EBPβ Tg/Tg/AEP^−/−^ mice. F) Kaplan‐Meier survival curves for male (left) and female (right) mice. Male, WT (n = 20); male, Thy 1‐C/EBPβ Tg/Tg (n = 15); male, Thy 1‐C/EBPβ Tg/Tg/AEP^−/−^ (n = 18); female, WT (n = 18); female, Thy 1‐C/EBPβ Tg/Tg (n = 14); female, Thy 1‐C/EBPβ Tg/Tg/AEP KO (n = 20). G,H) Individually graphed frailty phenotypes that significantly increase with aging, comparing WT, Thy 1‐C/EBPβ Tg/Tg and Thy 1‐C/EBPβ Tg/Tg/AEP^−/−^ mice for male and female. The number of animals assessed at each time point is shown as a row beneath each graph. I) Separately graphed (left) male and (right) female total FI scores during lifespan starting at the 1st month. Each dot is the total score of one animal at a specific age as indicated. n = all animals alive at each measurement time. All data are presented as the mean ± SEM from 3 to 6 independent experiments. *, P < 0.05; **, P < 0.01; ***, P < 0.001; ****, P < 0.0001.

**Figure 4:**
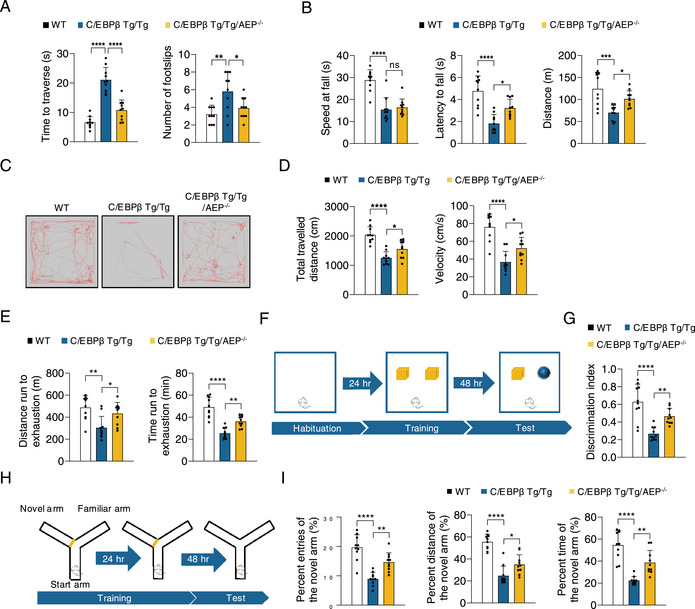
Knockout of AEP ameliorates age‐related motor and cognitive impairments caused by neuronal C/EBPβ overexpression in mice. A) Time to traverse and number of footslips in the beam walking test of WT, Thy 1‐C/EBPβ Tg/Tg, and Thy 1‐C/EBPβ Tg/Tg/AEP^−/−^ mice (n = 10). B) Latency to fall, distance, and speed at fall in accelerated rotarod test (n = 10). C) Representative traces in the open field test (n = 10). D) Total travelled distance and velocity in C). E) Duration and distance run until exhaustion in high‐intensity treadmill test (n = 10). F,G) Object recognition memory was assessed by novel object recognition NOR (n = 10). H,I) Spatial working memory was assessed using the Y maze (n = 10). All data are presented as the mean ± SEM from three to six independent experiments. *, P < 0.05; **, P < 0.01; ***, P < 0.001; ****, P < 0.0001.

To investigate whether C/EBPβ and AEP escalation in neurons mediate brain aging and longevity, we generated neuronal‐specific Thy 1‐C/EBPβ transgenic (Tg) mice and crossed them with AEP^−/−^ mice (Figure S2A,B, Supporting Information). IB and IF analysis validated the expression of C/EBPβ and AEP in the hippocampus from these mice (**Figure** **3**A–D; Figure S2C,D; Supporting Information). AEP enzymatic activities were confirmed by quantification assay (Figure S2E; Supporting Information). LE‐28, a fluorescent dye covalently conjugated to active AEP, analysis confirmed that AEP was distinctly activated in Tg/Tg brains compared to WT mice, which was entirely wiped out in Tg/Tg/AEP^−/−^ mice (Figure S2F,G, Supporting Information). We next monitored the age‐related phenotypes (Figure 3E). Remarkably, double Tg/Tg mice displayed a much shorter lifespan than wild‐type (WT) littermates, whereas knockout of AEP from these Tg/Tg mice significantly elongated the lifespan in both male and female mice (Figure 3F). Thy 1‐C/EBPβ mice demonstrated noticeable frailty indices versus WT mice, which was attenuated in Thy 1‐C/EBPβ/AEP^−/−^ mice (Figure 3G–I; Tables S1 and S2; Supporting Information). Beam walking test showed that the Tg/Tg mice took a longer time to traverse and more footslips than WT mice, and these defects were significantly alleviated in Tg/Tg/AEP^−/−^ mice (**Figure** **4**A). WT mice performed much better in latency to fall, distance, and speed at fall in accelerated rotarod test than Tg/Tg mice. Again, deletion of AEP from Tg/Tg mice significantly restored the endurance physical capability (Figure 4B). Open field test indicated Tg/Tg mice walking velocities were clearly slower and distances were shorter than WT mice, suggestive of weak physical activities (Figure 4C,D). Endurance behavioral test revealed that WT mice exhibited much longer duration and distance run until exhaustion in high‐intensity treadmill test than Tg/Tg mice (Figure 4E). The novel object recognition (NOR) indicated WT mice displayed much better memory than Tg/Tg mice. These abnormal behaviors were significantly relieved when AEP was deleted from Tg/Tg mice (Figure 4F,G). The spatial working memory in the Y maze indicated that Tg/Tg mice displayed much poorer memory than WT mice, which was partially restored in Tg/Tg/AEP^−/−^ mice (Figure 4H,I). Hence, neuronal C/EBPβ overexpression promotes functional decline and elicits short lifespan in young mice, and AEP deletion greatly attenuates the age‐related phenotypes.

### 2.4 AEP Cleaves NAMPT at N136 and Severely Impairs NAD^+^ Biosynthesis

**Figure 5:**
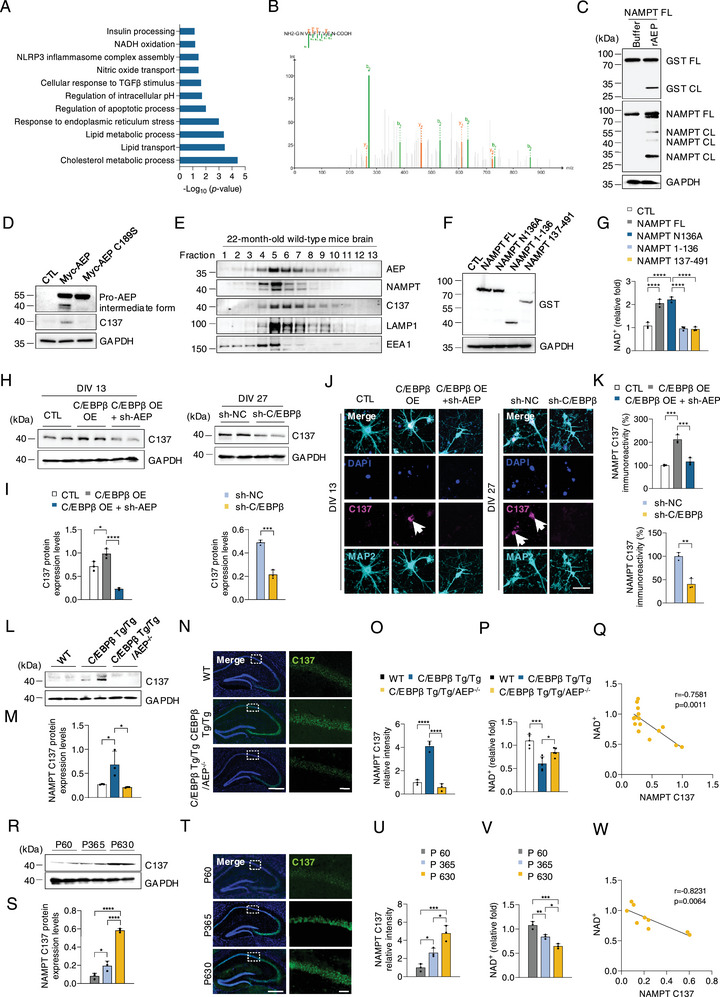
AEP cleaves NAMPT at N136 and severely impairs NAD^+^ biosynthesis. A) Top 11 KEGG pathway terms gathering the most DEGs in Thy 1‐C/EBPβ Tg/Tg mice versus WT mice as determined by enrichment analysis. B) Pull‐down assays from Thy 1‐C/EBPβ Tg/Tg mice brain homogenates using NAMPT antibody. LC/MS/MS analysis identified N136 as the major cutting site on NAMPT. C) HEK293 cells lysates overexpressing GST‐NAMPT were incubated with AEP for 20 min. Cleavage of purified GST‐NAMPT analyzed by immunoblotting. D) Cells co‐transfected with GST‐NAMPT and Myc‐AEP WT or Myc‐AEP C189S. Western blot showing that WT AEP but not C189S mutant AEP cleaved GST‐NAMPT. E) AEP and NAMPT fragments distribution in the subcellular fractions. Brain samples from WT mice were homogenated and fractionated on a discontinuous sucrose gradient. The fractions were analyzed by western blotting for AEP, NAMPT fragments, EEA1 (endosome marker), and LAMP1 (lysosomal marker). F) Western blot analysis of GST truncation in HEK293 cells lysates overexpressing GST‐NAMPT FL, NAMPT N136A, NAMPT 1–136, NAMPT 137–491. G) NAD^+^ levels were measured in F) (n = 3). H) Western blot analysis of NAMPT C137 levels in primary neurons (DIV 13) infected with LV‐CTL, LV‐C/EBPβ, or LV‐C/EBPβ + LV‐sh‐AEP and primary neurons (DIV 27) infected with LV‐sh‐NC or LV‐sh‐C/EBPβ. I) Quantification of protein levels in H). J) IF staining of MAP2 and NAMPT C137 in the indicated group (n = 3). Scale bars, 50 µm. K) Quantification of relative fluorescence intensity in J). L) Western blot analysis of NAMPT C137 levels in the hippocampus from WT, Thy 1‐C/EBPβ Tg/Tg, Thy 1‐C/EBPβ Tg/Tg/AEP^−/−^ mice (n = 3). M) Quantification of protein levels in L). N) IF staining of NAMPT C137 in the hippocampus from WT, Thy 1‐C/EBPβ Tg/Tg, Thy 1‐C/EBPβ Tg/Tg/AEP^−/−^ mice (n = 3). Scale bars, 500 µm, left, 50 µm, right. O) Quantification of relative fluorescence intensity in N). P) NAD^+^ levels in the hippocampus from WT, Thy 1‐C/EBPβ Tg/Tg, Thy 1‐C/EBPβ Tg/Tg/AEP^−/−^ mice (n = 3). Q) Negative correlation between NAD^+^ levels and NAMPT C137 fragments among WT, Thy 1‐C/EBPβ Tg/Tg, Thy 1‐C/EBPβ Tg/Tg/AEP^−/−^ mice. r and p values of the Pearson correlation coefficient are shown. R) Western blot analysis of NAMPT C137 levels in the hippocampus from different ages (60, 630 days postpartum) of C57BL/6 mice (n = 3). S) Quantification of protein levels in R). T) IF staining of NAMPT C137 in the hippocampus from different ages of C57BL/6 mice (n = 3). Scale bars, 500 µm, left, 50 µm, right. U) Quantification of relative fluorescence intensity in T). V) NAD^+^ levels in the hippocampus from different ages of C57BL/6 mice (n = 3). W) Negative correlation between NAD^+^ levels and NAMPT C137 fragments among different ages of C57BL/6 mice. r and p values of the Pearson correlation coefficient are shown. All data are presented as the mean ± SEM from three to six independent experiments. *, P < 0.05; **, P < 0.01; ***, P < 0.001; ****, P < 0.0001.

To explore the molecular mechanism underlying these motor and cognitive defects in Thy 1‐C/EBPβ Tg/Tg mice, we performed proteome analysis using hippocampal tissues of C/EBPβ Tg/Tg mice and their littermates. A total of 173 of 5983 proteins were upregulated or downregulated in Tg/Tg mice (threshold, log2 fold change > 0.3 or < −0.3 and p‐value < 0.05). Proteomic analysis indicates the significant differences in the proteins involved in the NADH oxidation, inflammation changes, and lipid metabolic process (**Figure** **5**A). The NAD^+^/NADH redox ratio directly reflects NAD^+^ levels.^[^ ^30^ ^]^ NAD^+^ concentrations change during aging, and modulation of NAD^+^ production can prolong both healthspan and lifespan. In the mammalian NAD^+^ metabolism, NAMPT is the rate‐limiting enzyme that catalyzes NAD^+^ biosynthesis from nicotinamide.^[^ ^28^ ^]^ To investigate whether C/EBPβ/AEP signaling affects NAD^+^ content by acting on NAMPT, we enriched the proteins from the brains of Thy 1‐C/EBPβ Tg/Tg mice and their littermates with NAMPT antibodies. LC/MS/MS detected NAMPT N136 residue in Thy 1‐C/EBPβ Tg/Tg mice (Figure 5B). Of note, N136 was highly conserved across diverse species (Figure S3A, Supporting Information). To further confirm that AEP as an enzyme, is responsible for NAMPT proteolytic truncation, we conducted an in vitro cleavage assay with purified GST‐NAMPT recombinant proteins in HEK293 cells, IB analysis showed that NAMPT was cleaved in the presence of AEP, revealed by both anti‐NAMPT and anti‐GST antibodies (Figure 5C). We then generated a rabbit polyclonal antibody that specifically recognized C137 in NAMPT to see whether NAMPT N136 residue is the direct product of AEP truncating NAMPT. After affinity purification, NAMPT C137 antibody selectively labeled the fragmented (137–491) but not full‐length (FL) NAMPT by IB analysis (Figure S3B, Supporting Information). The antibody specificity was further corroborated by IF staining. Anti‐C137 antibody specifically recognized the truncated NAMPT fragment in aged wild‐type mouse brains, which was totally abolished by preincubation with the antigen peptide (aa. 137–491 from NAMPT) but not the control peptide (Figure S3C, Supporting Information). AEP is a cysteine protease with C189 as the key active residue. Co‐transfection demonstrated that GST‐NAMPT was evidently cleaved by Myc‐AEP, producing 137–491 fragment, and this fragment disappeared in dominant‐negative C189S mutant transfected cells, underscoring that AEP is accountable for cutting NAMPT into 137–491 fragments (Figure 5D). To explore how a lysosomal protease AEP cleaves NAMPT, we investigated the subcellular basis for the interaction between AEP and NAMPT in the brain. Brain samples from aged WT mice were fractionated by differential centrifugation on a sucrose discontinuous gradient to separate cellular organelles. AEP was enriched in fractions 5 and 6, co‐fractionated with early endosomal entigen 1 (EEA1), a specific marker for the endosome, and lysosomal‐associated membrane protein 1 (LAMP1), a specific lysosomal marker. Moreover, AEP overlapped with NAMPT fragment in fractions 5 and 6 together with EEA1 and LAMP1, suggesting that AEP cleaves NAMPT in the endo‐lysosomal system (Figure 5E; Figure S3D, Supporting Information). Next, we detected NAD^+^ concentrations with HEK293 cells and found that both NAMPT full‐length and N136A mutant strongly mediated NAD^+^ biosynthesis as compared to N316 truncated N‐terminal or C‐terminal fragments (Figure 5F,G). Hence, AEP cuts NAMPT at N316 residue and impairs its enzymatic activity, diminishing NAD^+^ biosynthesis.

### 2.5 C/EBPβ/AEP Escalation in the Neuron Triggers NAMPT Fragmentation and NAD^+^ Decline

It is possible that NAMPT in neurons is truncated and dysfunctional. To test this notion, we reperformed IB with primary hippocampal neurons and found that NAMPT C137 levels were enhanced with C/EBPβ expression in young neurons (DIV 13), and depletion of AEP greatly reduced NAMPT C137 accumulation. On the other hand, deletion of C/EBPβ from aging neurons (DIV 27) evidently reduced NAMPT C137 fragments (Figure 5H,I). IF co‐staining with MAP2 and NAMPT C137 also exhibited a similar pattern, suggesting that C/EBPβ/AEP promotes neuronal senescence via truncating NAMPT (Figure 5J,K). To investigate whether NAMPT is also cleaved by elevated AEP in Thy 1‐C/EBPβ Tg/Tg mice, we performed IB and IF analysis and found that NAMPT C137 was elevated in the hippocampus from Thy 1‐C/EBPβ Tg/Tg mice, as compared to WT mice, and AEP deletion greatly prevented this cleavage (Figure 5L–O, Supporting Information). Notably, NAD^+^ was significantly reduced in Thy 1‐C/EBPβ Tg/Tg mice, which was restored in Thy 1‐C/EBPβ Tg/Tg/AEP^−/−^ mice, inversely coupled with NAMPT C137 fragments (Figure 5P,Q). IB and IF analysis of lysates from different ages of mouse brains showed truncated NAMPT C137 signals progressively elevated in the hippocampus (Figure 5R–U), inversely coupled with progressive NAD^+^ decrease (Figure 5V,W), suggesting that active AEP robustly cleaves NAMPT at N136 site and impairs NAD^+^ biosynthesis in the aging process.

### 2.6 Knockdown of C/EBPβ or AEP Restored NAD^+^ Levels and Attenuates Age‐Related Dysfunction in Aged Mice

To further interrogate the roles of C/EBPβ/AEP signaling in the aging process, we employed aged WT and C/EBPβ^+/−^ and AEP^+/−^ mice (Figures S4A and S5A, Supporting Information). We found that NAMPT C137 levels were markedly reduced in the brains of C/EBPβ^+/−^ and AEP^+/−^ mice (Figures S4B–E and S5B–E Supporting Information). Consequently, NAD^+^ concentrations were increased in comparison to aged WT littermates (Figures S4F and S5F, Supporting Information). Open field behavioral tests supported that knockdown either C/EBPβ or AEP substantially alleviated crippled physical activities (Figures S4G,H and S5G,H, Supporting Information). Spatial working memory Y‐maze and NOR assays also demonstrated that cognitive functions were improved in C/EBPβ^+/−^ and AEP^+/−^ mice as compared to aged WT mice (Figures S4I,J and S5I,J, Supporting Information). Hence, partially deleting C/EBPβ or AEP evidently boosts NAD^+^ levels and attenuates age‐related dysfunction.

### 2.7 Blockade of NAMPT Cleavage by AEP Rescues the Age‐Related Dysfunction caused by Neuronal C/EBPβ Overexpression in Mice

**Figure 6:**
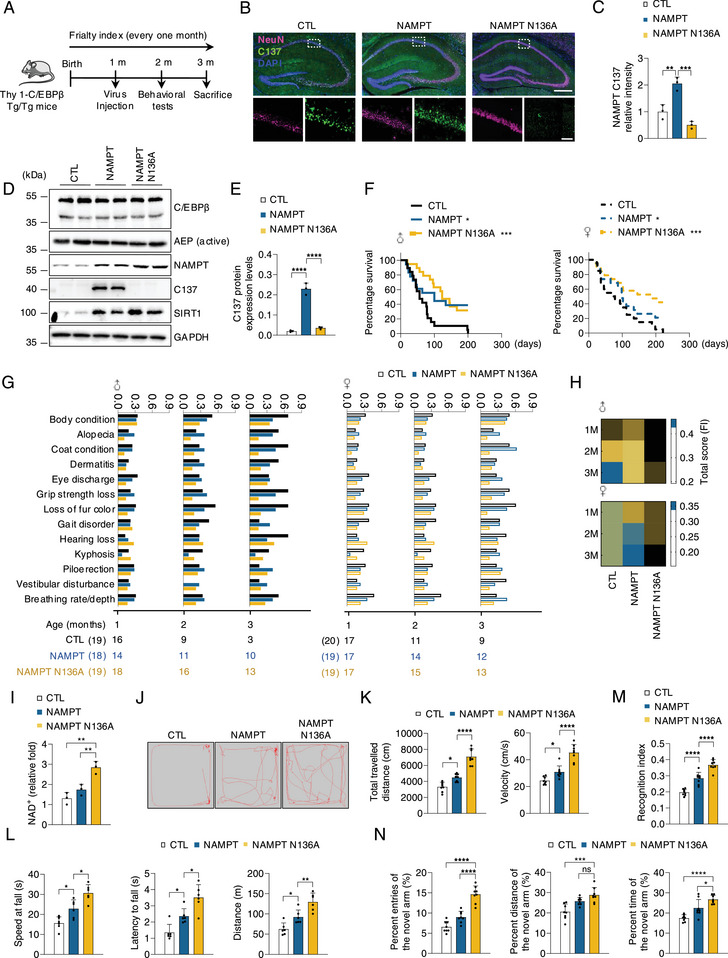
Blockade of NAMPT cleavage by AEP rescues the age‐related dysfunction caused by neuronal C/EBPβ overexpression in mice. A) The timeline of rAAV‐hsyn‐control, rAAV‐hsyn‐NAMPT, and rAAV‐hsyn‐NAMPT N136A administration to Thy 1‐C/EBPβ Tg/Tg mice. B) IF staining of NeuN and NAMPT C137 in the hippocampus from the indicated group (n = 3). Scale bars, 500 µm, top, 50 µm, bottom. C) Quantification of relative fluorescence intensity in B). D) Western blot analysis of C/EBPβ, AEP, NAMPT, NAMPT C137, and SIRT1 in the hippocampus (n = 3). E) Quantification of protein levels in D). F) Kaplan‐Meier survival curves for male (left) and female (right) mice. Male, CTL (n = 19); male, AAV‐NAMPT (n = 18); male, AAV‐NAMPT N136A (n = 19); female, CTL (n = 20); female, AAV‐NAMPT (n = 19); female, AAV‐NAMPT N136A (n = 19). G,H) Individually graphed frailty phenotypes that significantly increase with aging, comparing Thy 1‐C/EBPβ Tg/Tg mice injected with rAAV‐hsyn‐control, rAAV‐hsyn‐NAMPT, and rAAV‐hsyn‐NAMPT N136A for male and female. The number of animals assessed at each time point is shown as a row beneath each graph. I) NAD^+^ levels in the indicated group (n = 3). J) Representative traces in the open field test (n = 8). K) Total travelled distance and velocity in J). L) Latency to fall, distance, and speed at fall in accelerated rotarod test (n = 8). M) Object recognition memory was assessed by novel object recognition NOR (n = 8). N) Spatial working memory was assessed using the Y maze (n = 8). All data are presented as the mean ± SEM from 3 to 6 independent experiments. *, P < 0.05; **, P < 0.01; ***, P < 0.001; ****, P < 0.0001.

To examine whether AEP‐mediated NAMPT N136 cleavage is accountable for the impaired motor and cognitive behaviors and short lifespan, we injected AAV virus expressing NAMPT or AEP‐resistant N136A mutant into the hippocampus of Thy 1‐C/EBPβ Tg/Tg mice at 1 month of age, and monitored the performance of mice in two months (**Figure** **6**A). IF and IB analysis showed that NAMPT C137 proteolytic cleavage was increased from control to mice injected AAV‐NAMPT and was blocked by NAMPT N136A mutant injection (Figure 6B–E; Figure S6A, Supporting Information). NAMPT FL levels were elevated in NAMPT overexpressed mice versus control mice, and even more in NAMPT N136A mutant injected mice (Figure 6D; Figure S6A, Supporting Information). Subsequently, SIRT 1 levels echoed with NAMPT FL patterns (Figure 6D; Figure S6A, Supporting Information). It was noteworthy that SIRT1 level was significantly enhanced when NAMPT cleavage was blocked. Survival analysis showed that both NAMPT and N136A mutant significantly elongated the longevity of Tg/Tg mice compared to control virus, with the latter better than the former (Figure 6F). Frailty analysis suggested that the indices were significantly improved in both AAV‐NAMPT and AAV‐NAMPT N136A mice versus control mice, with AAV‐NAMPT N136A mutant injection even better (Figure 6G,H; Figure S6B, Supporting Information). As expected, NAD^+^ concentrations were substantially increased in AEP‐resistant N136A expressed mice as compared to control and AAV‐NAMPT injected mice (Figure 6I). Beam walking test, open field test, rotarod test, and endurance behavioral test indicated overexpression of NAMPT or N136A mutant significantly improved the exercise ability in comparison to control mice, with AEP‐resistant mutant greater than NAMPT (Figure 6J–L; Figure S6C,D, Supporting Information). Spatial working memory Y‐maze and NOR assays revealed that both NAMPT and N136A mice exhibited improved spatial memory, with the latter better than the former (Figure 6M,N). Hence, blockage of NAMPT N136 cleavage by AEP restores NAD^+^ and SIRT1 levels in Thy 1‐C/EBPβ Tg/Tg mice, increasing the lifespan and improving physical and cognitive functions.

### 2.8 Inhibition of AEP with Specific Inhibitor #11a Rescues the Age‐Related Dysfunction Caused by Neuronal C/EBPβ Overexpression in *C. elegans* and Mice

To test whether pharmacological supplementation of NAD^+^ or inhibition of AEP prevents neuronal senescence caused by C/EBPβ overexpression. C/EBPβ was overexpressed in young primary hippocampal neurons (DIV 10) using AAV, and the neurons were subsequently treated with vehicle, #11a, NR, or NMN. Cellular senescence markers were evaluated at DIV 13. IB assays revealed that NMN failed to block NAMPT C137 truncation, whereas #11a robustly inhibited active AEP and decreased NAMPT C137 fragmentation (Figure S7A,B, Supporting Information). In comparison to the vehicle, #11a, NR, and NMN significantly decreased the accumulation of β‐gal signals, mitigated the decline in mitochondrial membrane potential, and reduced the expression of inflammatory factors associated with elevated NAD^+^ levels. Among them, #11a demonstrated the most pronounced efficacy (Figure S7C–H, Supporting Information). We further isolated primary neurons from Thy 1‐C/EBPβ Tg/Tg mice and their littermates, and assessed cellular senescence markers in DIV 13. IB assays revealed that C/EBPβ and AEP were greatly enhanced in neurons from Thy 1‐C/EBPβ Tg/Tg mice in comparison with WT mice, with NAMPT C137 fragmentation (Figure S8A,B, Supporting Information). Senescence assays revealed that overexpression of C/EBPβ significantly enhanced β‐galactosidase staining, upregulated the expression of inflammatory factors, and diminished mitochondrial membrane potential, which was closely associated with a reduction in NAD^+^ levels (Figure S8C–H, Supporting Information). We then treated neurons from Thy 1‐C/EBPβ Tg/Tg mice with vehicle, #11a, NR, and NMN at DIV 10 and examined cellular senescence markers at DIV 13. IB assays revealed that NMN failed to block NAMPT C137 truncation, whereas #11a robustly inhibited active AEP and decreased NAMPT C137 fragmentation (Figure S8I,J, Supporting Information). Senescence test revealed that #11a, NR, and NMN significantly inhibited the C/EBPβ overexpression‐induced cellular senescence phenotypes, with #11a demonstrating the most pronounced effect (Figure S8K–P, Supporting Information). Therefore, supplementation of NAD^+^ or inhibition of AEP could prevent neuronal senescence induced by C/EBPβ overexpression at the cellular level.

**Figure 7:**
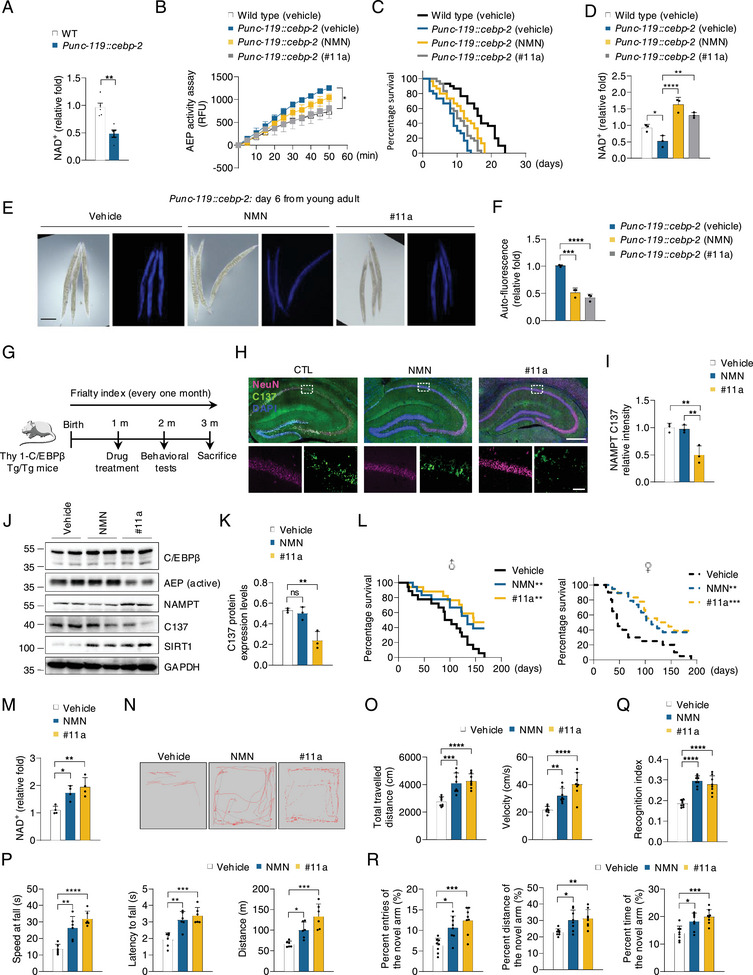
Inhibition of AEP with specific inhibitor #11a rescues the age‐related dysfunction caused by neuronal C/EBPβ overexpression in mice and *C.elegans*. A) Relative NAD^+^ levels in wild type (N2) or *unc‐119::cebp‐2* adult worms (n = 3). B) Quantification of NMN and #11a effects in AEP protease activities. Day two worm's protein lysates were collected for AEP activity assay (n = 3). C). Kaplan‐Meier survival curves of wild type (N2) or *unc‐119::cebp‐2* worms treated with vehicle (0.1% DMSO), NMN (1 mM in 0.1% DMSO), or #11a (1 mM in 0.1% DMSO) at 25 °C. D) Relative NAD^+^ levels in the indicated group (n = 3). E) At day 6 post, young adult at 25 °C, lipofuscin was measured by assessing auto‐fluorescence (via a DAPI filter set). Representative images of each group of worms are shown. Scale bars, 100 µm. F) Quantification of relative fluorescence intensity in E). G) The timeline of NMN or #11a treatment to Thy 1‐C/EBPβ Tg/Tg mice. H) IF staining of NeuN and NAMPT C137 in the hippocampus from the indicated group (n = 3). Scale bars, 500 µm, top, 50 µm, bottom. I) Quantification of relative fluorescence intensity in H). J) Western blot analysis of C/EBPβ, AEP, NAMPT, NAMPT C137, and SIRT1 in the hippocampus (n = 3). K) Quantification of protein levels in J). L) Kaplan‐Meier survival curves for male (left) and female (right) mice. Male, CTL (n = 18); male, NMN (n = 18); male, #11a (n = 17); female, CTL (n = 20); female, NMN (n = 19); female, #11a (n = 18). M) NAD^+^ levels in the indicated group (n = 4). N) Representative traces in the open field test (n = 8). O) Total travelled distance and velocity in N). P) Latency to fall, distance, and speed at fall in accelerated rotarod test (n = 8). Q) Object recognition memory was assessed by novel object recognition NOR (n = 8). R) Spatial working memory was assessed using the Y maze (n = 8). All data are presented as the mean ± SEM from 3 to 6 independent experiments. *, P < 0.05; **, P < 0.01; ***, P < 0.001; ****, P < 0.0001.

To explore the regulation of longevity, we used *C. elegans*, a well‐established model system for studying aging. Transgenic nematodes with a WT background were created that expressed *cebp‐2* under the control of the pan‐neuronal promoter from *unc‐119*. As expected, NAD^+^ concentrations were substantially decreased in *unc‐119::cebp‐2* lines comparable to N2 WT worms (**Figure** **7**A). The major enzymes involved in NAD^+^ synthesis were dramatically decreased in *unc‐119::cebp‐2* lines, which was negatively correlated with mRNA levels of *cebp‐2 and lgmn‐1* (Figure S9A, Supporting Information). To test whether NMN or #11a attenuates the short lifespan and frailty associated with *unc‐119::cebp‐2* worms, we treated WT and *unc‐119::cebp‐2* worms with vehicle, NMN, or #11a. AEP activities were substantially blocked by #11a (Figure 7B). Both NMN and #11a significantly elongated the lifespan of *unc‐119::cebp‐2* worms, associated with markedly escalated NAD^+^ levels (Figure 7C,D). Moreover, these compounds also decreased auto‐fluorescence, as a measure of senescence in *C. elegans* (Figure 7E,F).

Next, we treated one month old Thy 1‐C/EBPβ Tg/Tg mice with 300 mg k^−1^g NMN or 7.5 mg k^−1^g #11a orally in Thy 1‐C/EBPβ Tg/Tg mice (Figure 7G). In vitro AEP enzymatic activity assay and Ex vivo AEP enzymatic assay with LE28 revealed that AEP was potently blocked by #11a but not NMN in the brains of Thy 1‐C/EBPβ Tg/Tg mice (Figure S10A–C, Supporting Information). IF and IB assays revealed that NMN failed to block NAMPT C137 truncation, whereas #11a robustly inhibited active AEP and decreased NAMPT C137 fragmentation, resulting in FL NAMPT and SIRT1 upregulation (Figure 7H–K; Figure S10D, Supporting Information). Survival analysis showed that both chemicals pronouncedly elongated the lifespan of Thy 1‐C/EBPβ Tg/Tg mice as compared to vehicle‐treated mice, associated with elevated NAD^+^ levels, and #11a performed better than NMN (Figure 7L,M). Frailty assay showed that NMN or #11a significantly alleviated the aging indices, with the latter better than the former (Figure S10E–G, Supporting Information). Behavioral tests demonstrated that these drugs substantially improved the frail physical activities and impaired cognitive functions (Figure 7N–R; Figure S10H,I, Supporting Information). Together, these results strongly support that AEP inhibitor #11a displays a better anti‐aging therapeutic effect than NMN.

## 3 Discussion

The brain demonstrates extensive pathophysiological changes in its constituent cells, structures, and functions during aging. In this study, we show that C/EBPβ/AEP signaling is gradually escalated in the hippocampal neurons in an age‐dependent manner, tightly correlating with elevated neuro‐inflammation and oxidative stress. This pathway activation is closely coupled with progressive neuronal loss during aging in the brains of both humans and mice (Figure 1). These observations are consistent with a previous report that bioinformatic analysis reveals numerous key genes in aging brains in SAMP8 mice, indicating that C/EBPβ, IL‐6, IL‐1β, and brain‐derived neurotrophic factor etc., play important roles in this process.^[^ ^31^ ^]^ In primary neuronal cultures, C/EBPβ overexpression elicits prominent neuronal senescence and mitochondrial dysfunctions and abundant inflammation, which are greatly diminished when AEP is deleted (Figure 2). Accordingly, AEP is highly upregulated in Thy 1‐C/EBPβ Tg/Tg mice, promotes functional decline, and elicits a short lifespan in young mice. These age‐related phenotypes are completely abolished when AEP is knocked out in Thy 1‐C/EBPβ Tg/Tg mice, indicating that these events are primarily mediated by AEP protease (Figures 3 and 4). Notably, AEP pronouncedly cuts NAMPT at N136 residue, abolishing NAD^+^ synthesis (Figure 5). These findings are consistent with previous findings that neuronal NAMPT deletion causes mitochondrial dysfunction, progressive loss of body weight, motor neuron degeneration, motor function deficits, and death.^[^ ^32^ ^]^ Consequently, Thy 1‐C/EBPβ Tg/Tg mice exhibit frail physical activity compared to WT mice, and depletion of AEP significantly relieves these defects (Figure 4). NAMPT is essential in the hippocampal and cortical excitatory neurons’ role in cognition.^[^ ^22^ ^]^ Similarly, Thy 1‐C/EBPβ Tg/Tg mice display cognitive deficits that are alleviated when AEP is knocked out (Figure 4). It is worth noting that NAMPT is steadily fragmented during brain aging, associated with NAD^+^ decline. These findings are further supported by immunoblotting analysis of the brains from Thy 1‐C/EBPβ Tg/Tg/AEP^−/−^ mice (Figure 5).

Structurally, we also found NAMPT/NAD^+^/SIRT1 signaling is prominently upregulated in Thy 1‐C/EBPβ Tg/Tg mice via AEP‐resistant NAMPT N136A overexpression (Figure 6). SIRT1 promotes neurite outgrowth and axon development^[^ ^33^ ^]^ and regulates dendritic arborization, long‐term potentiation, and learning and memory.^[^ ^34^, ^35^ ^]^ Downregulation of SIRT1 in the liver of old mice is mediated by C/EBPβ‐histone deacetylase 1 complexes, which bind to and repress the SIRT1 promoter, resulting in impaired body homeostasis and inhibition of liver proliferation.^[^ ^36^, ^37^ ^]^ These observations fit with our results that SIRT1 is age‐dependently repressed, inversely coupled with an escalation of C/EBPβ in the hippocampus, and deletion of C/EBPβ elevates SIRT1 (Figure 1). Brain aging also affects the functions of peripheral tissues and organs through many hormones and the autonomic nervous system. In particular, the hypothalamus plays a critical part in the production of many hormones and in the regulation of the autonomic nervous system, and it is emerging that age‐associated decline in hypothalamic function mediates aging at a systemic level and ultimately affects longevity.^[^ ^1^ ^]^ For instance, hypothalamic knockout of SIRT1 modulates various metabolic events in peripheral organs, including brown adipose tissue remodeling, insulin sensitivity.^[^ ^38^, ^39^ ^]^ Therefore, it is likely that the brain via Sirtuin signaling is an important part of a network that regulates aging throughout the body. Mounting evidence supports that Sirtuins contribute to age‐associated cognitive decline through the regulation of synaptic plasticity and adult neurogenesis.^[^ ^40^ ^]^ SIRT1‐deficient mice have impaired hippocampal‐dependent memory, associated with decreased long‐term potentiation in the hippocampal CA1 region.^[^ ^35^, ^40^ ^]^ Sirtuins interact with other pathways that also contribute to the control of aging, such as the insulin‐FOXO and mechanistic target of rapamycin pathways, in many cellular contexts.^[^ ^41^, ^42^, ^43^ ^]^ Interestingly, AEP‐resistant NAMPT N136A overexpression restores SIRT1 in the hippocampus of Thy 1‐C/EBPβ Tg/Tg mice, correlating with NAD^+^ levels augmentation. Accordingly, the lifespan and cognitive functions are significantly augmented (Figure 6).

In worms, NAD^+^ consumer enzymes hydrolyze NAD^+^ and generate NAM that is recycled to reform NAD^+^ via pathways that differ between invertebrates and vertebrates. However, administration of NAD^+^ or AEP inhibitor #11a significantly alleviates cognitive dysfunctions and elongates the life expectancy in worms (Figure 7; Figure S8, Supporting Information). Consistently, administration of NMN or #11a that blocks NAMPT proteolytic fragmentation substantially increases NAD^+^ concentrations and elongates the life expectancy of Thy 1‐C/EBPβ Tg/Tg mice, accompanied by amelioration of cognitive deficits (Figure 7; Figure S9, Supporting Information). It remains unclear why NMN inhibits AEP enzymatic activities in *unc‐119::cebp‐2* worms but not in the brains of Thy 1‐C/EBPβ Tg/Tg mice. Presumably, NMN brain exposure is modest in the mouse brain due to the brain‐blood barrier. In agreement with the pathological roles of neuronal C/EBPβ/AEP signaling in aging and AD pathogenesis, nicotinamide ribose (NR) ameliorates cognitive impairments in aged mice and AD mouse models.^[^ ^44^ ^]^ NMN also relieves cognitive impairment and amyloid deposition in AD mouse models.^[^ ^16^, ^17^ ^]^ Hence, NAMPT may exert these beneficial effects via NAD^+^‐activated SIRT1, consistent with previous reports that NAMPT enzymatic activity stimulates the synthesis of NAD^+^, which is an essential cofactor of SIRT deacetylases, partially explaining why #11a performs a better anti‐aging effect than NMN.^[^ ^40^ ^]^

**Figure 8:**
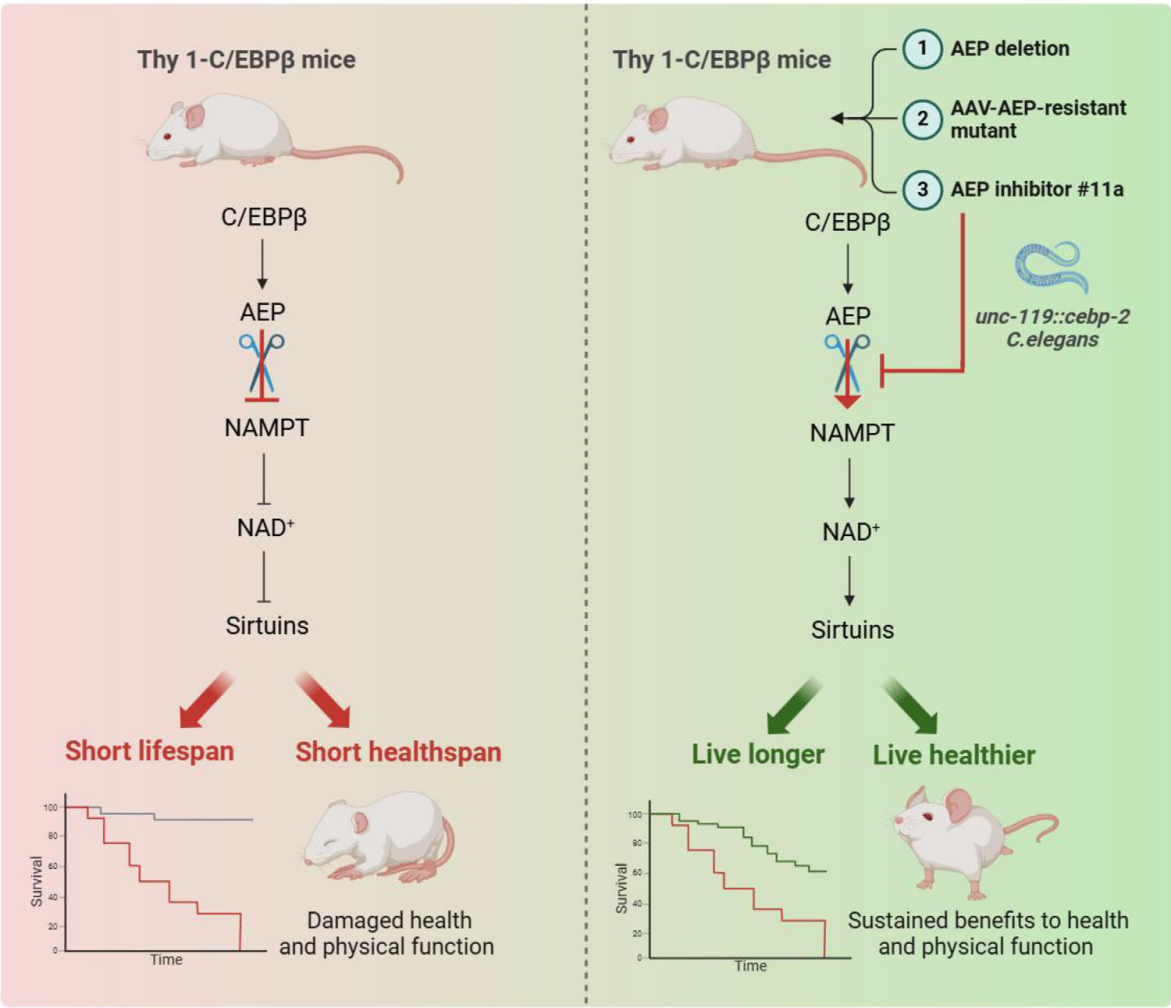
Schematic diagram illustrating the mechanism of shortened lifespan in Thy 1‐C/EBPβ transgenic mice and intervention strategies. Briefly, C/EBPβ/AEP escalation in the aging brain triggers NAMPT fragmentation and NAD^+^ depletion. Knockout of AEP from Thy 1‐C/EBPβ Tg/Tg mice ameliorates cognitive impairments and elongates the lifespan. AEP‐resistant NAMPT N136A mutant rescues the cognitive dysfunction and short lifespan of Thy 1‐C/EBPβ Tg/Tg mice. AEP inhibitor #11a administration alleviates cognitive deficits and life expectancy of Thy 1‐C/EBPβ Tg/Tg mice and *C. elegans*.

Therefore, the finding that neuronal C/EBPβ escalation impairs physical and cognitive functions and shortens lifespan via inactivating NAMPT/NAD^+^/SIRT1 pathway that is evolutionarily conserved from *C. elegans* to mammals (**Figure** **8**). Conceivably, blockage of the major C/EBPβ downstream AEP via its specific inhibitor may provide an unprecedented strategy for fighting aging and various age‐associated diseases.

## 4 Experimental Section

### Mice

Wild‐type C57BL/6J mice were ordered from the Jackson Laboratory (000664). C/EBPβ knockout mice had been described.^[^ ^45^ ^]^ Since the homozygous mutation was lethal on pure strain backgrounds, Cebpb mice were maintained as heterozygotes on two separate strain backgrounds (C57BL/6 and 129 Sv). The AEP knockout mice on a mixed 129/Ola and C57BL/6 background were generated as reported.^[^ ^46^ ^]^ Thy 1‐C/EBPβ mice were generated by Cyagen Company. Thy 1‐C/EBPβ mice were crossed with AEP^−/−^ mice to generate Thy 1‐C/EBPβ/AEP^−/−^ mice. Investigators were blinded to the group allocation during the animal experiments. All the mice were bred in specific pathogen‐free facilities at the Shenzhen Institutes of Advanced Technology (SIAT), Chinese Academy of Sciences (CAS), and maintained on a 12‐h light/dark cycle with unrestricted access to water and food. All animal experiments followed the guidelines of the Institutional Animal Care and Use Committee of Shenzhen Institutes of Advanced Technology, Chinese Academy of Sciences (Approval No. LLSQ2111110006).

### Human Brain Tissue Collection and Ethical Approval

Postmortem human brain samples for immunofluorescence (IF) staining were obtained from the Chinese Brain Bank Center (CBBC). Written informed consent was acquired from all participants or their legal representatives prior to sample collection. This study was approved by the Ethics Committee of the Chinese Brain Bank Center (Approval No. 2024‐scuec‐046). All procedures involving human tissues were conducted in strict compliance with the ethical guidelines of the Institutional Review Board (IRB) of Shenzhen Institutes of Advanced Technology, Chinese Academy of Sciences. Subject baseline characteristics for the samples are listed in Table S3 (Supporting Information). All tissues were fixed with 4% paraformaldehyde.

### Cells

HEK293 cells were grown in DMEM supplemented with 10% FBS and 1% Penicillin/Streptomycin (Pen/Strep) at 37 °C with 5% CO_2_.

### Primary Cultured Neurons

Briefly, primary cortical neurons were isolated from E18 Sprague‐Dawley rats. Brain tissues were dissected, dissociated, and incubated with 5 ml of D‐Hanks containing 0.25% trypsin for 5 min, centrifuged at 1000 g for 5 min after the addition of 4 ml of the neuronal plating medium containing Dulbecco's modified Eagle's medium/F12 with 10% fetal bovine serum. Then, the cells were resuspended, about 5 × 10^5^ cells were plated onto each well of 12‐well plates for Western blotting, and 1 × 10^5^ cells were plated onto each glass coverslip for cell imaging. The neurons were then put into a humidified incubator with 5% CO_2_ at 37 °C. The medium was changed to neurobasal medium supplemented with 2% B27 (maintenance medium) after 2 to 4 h. Neurons at 13 or 27 DIV were treated with viruses.

### Survival

Survival experiments were performed as previously described.^[^ ^47^ ^]^ The principle endpoint of the lifespan study was natural death. The age was recorded at which mice were found dead or selected for euthanasia (a procedure for mice deemed unlikely to survive for the next 48 h and are in enormous discomfort). The criteria for euthanasia were based on an independent assessment by a veterinarian, according to AAALAC guidelines, and only where the condition of the animal was considered.^[^ ^48^ ^]^ Severe lethargy, rapid weight loss (over two weeks > 20%), severe distended abdomen and body condition score with signs of pain (grimace), inability to move despite the stimuli, severe ulcer or bleeding tumor, severe temperature loss with abnormal breathing rate. Animals found dead or euthanized were necropsied for pathology score.

### Aging Index (Frailty Index)

Aging Index was performed as previously described.^[^ ^49^ ^]^ For the subjective properties of the assessments, all measurements were completely blinded. These assessments indicate age‐associated deterioration of health and include evaluation of the animal musculoskeletal system, the vestibulocochlear/auditory systems, the ocular and nasal systems, the digestive system, the urogenital system, the respiratory system, signs of discomfort, body weight, and body surface temperature. Zero was assigned if no sign of frailty is observed and the animal is healthy for that phenotype. A moderate phenotype and a severe phenotype will be scored 0.5 and 1, respectively.

### Endurance Testing of Mice

Mice were acclimatized to the treadmill system for three days prior to endurance testing by running at 10–15 mmin^−1^ for 20 min. The speed started at 5 mmin^−1^ for 5 min, and then the speed was increased by one every minute until 21 mmin^−1^ and kept constant. Mice were run until exhaustion and were considered exhausted when they sat on the shocker plate for more than 10 swithout attempting to reinitiate running.

### Exercise Training of Mice

Mice were acclimatized to the treadmill system for three days prior to the start of exercise training by running at 10 mmin^−1^ for 20 min. The animals that were successfully acclimated were then trained at 15–20 mmin^−1^ at 5° inclination for 30 min once daily for 30 days. Mice receiving treatment were given NMN (500 mg k^−1^g/day) and/or #11a (7.5 mg k^−1^g/day) throughout the course of the exercise training regimen. NMN given via drinking water was changed every three days and #11a given via food was changed weekly. The exhaustive exercise capacity of the mice was tested at the end of training.

### Maze

The Y Maze task was conducted as previously described.^[^ ^50^ ^]^ Briefly, during the training phase, the mice were placed into the start arm facing the wall and were allowed to explore the start and trained arm for 5 min, while the entry to the 3rd arm (novel arm) was blocked. The maze was cleaned between each mouse to remove odor cues, and the trained arm was alternated between mice. After training, the mouse was returned to its home cage. After 45 min, the mouse was returned to the start arm and was allowed to explore all three arms for 5 min. The number of entries and the time spent in each arm was quantified using the Smart Video Tracking Software (Panlab; Harvard Apparatus). The percentage of entries in each arm was defined as the number of entries in each arm divided by the total number of entries in all arms during the first minute of the task. The discrimination index was quantified by (novel arm−trained arm)/(novel arm + trained arm). Mice that did not perform three entries during the first minute of testing were excluded.

### Rotarod Assay

Rotarod assay was performed by placing each mouse on a horizontal rod, which rotates with an acceleration rate of five rounds per minute (rpm) to achieve a maximum of 40 rpm in 99 s. Latency to fall, distance, and speed at fall from the rod were recorded (with a cut‐off time of 4 min). Each mouse underwent three consecutive trials separated by a 20 min resting interval. Measures from the last two trials are averaged to determine the time before falling.

### Novel Object Recognition Test

Mice were presented with two identical objects during the first session, and then one of the two objects was replaced by a novel object during a second session. On day one, a habituation phase in an empty arena (for 5 min), was followed 24 h later by the training phase, which allowed for a 5 min exploration in the habituated arena in which two identical objects were placed in opposite quadrants. The testing phase followed a gap of 20 min. For testing, one object was replaced with a novel object, followed by 5 min of exploration.

### Open Field Test

The open field test measures hyperactivity through locomotion and anxious behavior. The open field box consisted of a square black box (40 cm x 40 cm x 40 cm). Each animal was placed in the box for ten minutes. The amount of time and distance traveled in the box (measured with videotrack) was measured.

### Virus Production and Delivery

rAAV‐hsyn‐hNAMPT, rAAV‐hsyn‐hNAMPT N136A viruses were purchased from the BrainVTA company. Thy 1‐C/EBPβ mice were injected with rAAV‐hsyn‐vectors, rAAV‐hsyn‐hNAMPT, and rAAV‐hsyn‐hNAMPT N136A virus in a volume of 100 µL via the tail veins. The titer of the virus was 2 × 10^13^ genomes per ml. Lentivirus (LV)‐C/EBPβ and LV‐shAEP virus were purchased from OBio company.

### In vitro NAMPT Cleavage Assay

To assess the cleavage of NAMPT by AEP in vitro, HEK293 cells were transfected with GST‐NAMPT plasmids by PEI. 48 h after transfection, the cells were collected, washed once in PBS, lysed in lysis buffer (50 mM sodium citrate, 5 mM dithiothreitol (DTT), 0.1% CHAPS, and 0.5% Triton X‐100, pH 7.4), and centrifuged for 10 min at 14000 g at 4 °C. The supernatant was then incubated with mouse kidney lysates at pH 7.4 or 6.0 at 37 °C for 30 min. To measure the cleavage of purified NAMPT fragments by AEP, GST‐ NAMPT was purified with glutathione beads. The purified NAMPT proteins were incubated with recombinant AEP (5 mgmL^−1^) in AEP buffer (50 mM sodium citrate, 5 mM DTT, 0.1% CHAPS, and 0.5% Triton X‐100, pH 6.0) for 5 to 15 min. The samples were then boiled in SDS loading buffer and analyzed by immunoblotting.

Generation of antibodies that specifically recognize the AEP‐generated NAMPT fragments. The anti‐NAMPT C137 antibodies were generated by immunizing rabbits with the peptide TDPECYWLTNWIETI. The antiserum was pooled, and the titers against the immunizing peptide were determined by ELISA. The maximal dilution giving a positive response with the chromogenic substrate for horseradish peroxidase was 1:50 000. The immunoactivity of the antiserum was further confirmed by Western blotting and immunohistochemistry.

### Mass Spectrometry Analysis

Protein samples were in‐gel digested with trypsin. Peptide samples were resuspended in loading buffer (0.1% formic acid, 0.03% trifluoroacetic acid and 1% acetonitrile) and loaded onto a 20‐cm nano‐high‐ performance liquid chromatography column (internal diameter 100 mm) packed with Reprosil‐Pur 120 C18‐AQ 1.9 mm beads and eluted over a 2 h 4–80% buffer B reverse phase gradient (buffer A: 0.1% formic acid and 1% acetonitrile in water; buffer B: 0.1% formic acid in acetonitrile) generated by a NanoAcquity UPLC system (Waters Corporation). Peptides were ionized with 2.0 kV electrospray ionization voltage from a nano‐ESI source (Thermo) on a hybrid LTQ XL Orbitrap mass spectrometer (Thermo). Data‐dependent acquisition of MS spectra at 120000 resolution (full width at half maximum) and tandem mass spectrometry (MS/MS) spectra were obtained in the Orbitrap after electron‐transfer dissociation with supplemental activation with high energy (EThcD) for peptide masses. To identify AEP‐cleavage sites in human NAMPT, Proteome Discoverer 2.0 (PD) was used to search and match MS/MS spectra to a complete human proteome database (NCBI reference sequence revision 62, with 68746 entries) with a ± 10‐ppm mass accuracy threshold and allowable cleavages at glutamates and asparagines. A percolator was used to filter the peptide spectral matches to a false discovery rate of <1%. All MS/MS spectra for putative AEP‐generated NAMPT cleavage sites were manually inspected.

### Genotyping PCR Analysis

A small piece of tail or tissue obtained from Thy 1‐C/EBPβ, Thy 1‐C/EBPβ/AEP^−/−^ and WT was incubated in 50 mL of alkaline lysis reagent (25 mM NaOH, 0.2 mM EDTA, pH 12) and incubated at 100 °C for 1 h. After cooling, 50 mL of neutralizing reagent (40 mM Tris‐HCl, pH 5) was added, and 2 mL of the supernatant was used for PCR to detect the excision of C/EBPβ or LGMN gene using the primers.

### RNA Analysis

Total mRNA was isolated from cells and tissues using TRIzol. cDNAs were synthesized from 1 mg of total RNA using iScript Reverse Transcription Supermix. qPCR was performed with LightCycler 48 SYBR Green I Mastermix using the LightCycler 480 System according to the manufacturer's instructions. Relative mRNA expression levels were calculated using the ΔΔC*_t_* method. Quantification of relative mRNA expression was normalized to the expression of Gapdh.

### Western immunoblotting

Cells and brain tissue were washed with ice‐cold PBS and lysed in 50 mM Tris‐HCl, pH 7.4, 40 mM NaCl, 1 mM EDTA, 0.5% Triton X‐100, 1.5 mM Na_3_VO_4_, 50 mM NaF, 10 mM sodium pyrophosphate and 10 mM sodium β‐glycerophosphate, supplemented with protease inhibitor cocktail at 4 °C for 0.5 h, and centrifuged for 25 min at 15000 rpm. The supernatant was boiled in SDS loading buffer. After SDS‐PAGE, the samples were transferred to a nitrocellulose membrane. The membrane was blocked with TBS containing 5% non‐fat milk and 0.1% Tween 20 (TBST) at room temperature for 2 h, followed by incubation with primary antibodies at 4 °C overnight, and with secondary antibodies at room temperature for 2 h. After washing with TBST, the membrane was developed using the enhanced chemiluminescent detection system.

### Immunofluorescence

Free‐floating 12 µm sections were used in immunostaining. For immunofluorescence staining, the sections were incubated overnight at 4 °C with primary antibodies. After washing with PBST, the sections were incubated with a mixture of Alexa Fluor 488‐, 555‐, and 647‐coupled secondary antibodies for detection. DAPI (1 µgmL^−1^) was used for staining nuclei. Last, coverslips were mounted on glass slides and imaged using a confocal microscope (LSM 980, Zeiss). The fluorescence density was quantified using the Image J software. The ratio of the average fluorescence intensity of each group to that of the control group was used to draw the histogram.

### SA‐β‐gal Assay

SA‐β‐gal assay was performed as previously described.^[^ ^51^ ^]^ Both cells and tissue explants were washed twice with PBS, then fixed for 5 min with 2% formaldehyde and 0.2% glutaraldehyde in PBS at room temperature, washed with PBS and then incubated overnight at 37 °C in staining solution with 40 mM citric acid NA phosphate, 5 mM K_4_Fe(CN)_6_, 5 mM K_3_Fe(CN)_6_ (Fluka analytical, 34 272), 150 mM NaCl, 2 mM MgCl_2_ and 1 mgml^−1^ X‐Gal (Roche, R0404) in water. The samples were washed twice with PBS before imaging by microscopy (Zeiss Axio Vert.A1).

### Assessing AEP Activity In Vivo (LE‐28 signal detection)

The probe of LE‐28 was kindly provided by Dr. Edgington‐Mitchell from the University of Melbourne, Australia. LE‐28 (2 mg k^−1^g) was injected into mice via the tail vein. Mice were anesthetized with isoflurane and placed in the chamber of IVIS 100 system at 4 h after injection. AEP activity was monitored by fluorescence produced by LE‐28 and took images.

### AEP Activity Assay

Tissue homogenates or cell lysates (10 µg) were incubated in 200 µl assay buffer (20 mM citric acid, 60 mM Na_2_HPO_4_, 1 mM EDTA, 0.1% CHAPS, and 1 mM dithiothreitol, pH 6.0) containing 20 µM δ‐secretase substrate, Z‐Ala‐Ala‐Asn‐AMC (Bachem). AMC released by substrate cleavage was quantified by measuring at 460 nm using a fluorescence plate reader at 37 °C in kinetic mode.

### NAD^+^/NADH Assay

Levels of NAD^+^ in neurons and muscle homogenates were measured using a commercially available kit. Alternatively, NAD^+^ levels in muscle were measured by an assay in‐house developed method.^[^ ^52^ ^]^ In brief, muscle samples were homogenized in extraction buffer (10 mM Tris‐HCl, 0.5% Triton X‐100, 10 mM Nicotinamide, pH 7.4) and then centrifuged (12000 x g for 5 min at 4 °C), after which an aliquot of supernatant was taken for protein quantification. After phenol:chloroform:isoamylalcohol (25:24:1) and chloroform extractions, the supernatant was separated in two aliquots. One was used to measure total NAD. The other aliquot was acidified with HCl, and then neutralized with NaOH on ice to quantify NAD^+^. Samples were mixed in a 96‐well plate, samples were mixed with alcohol dehydrogenase at room temperature. Total NADH and NAD^+^ were quantified using a plate reader.

### *C*. *Elegans* Experiments

*C. elegans* strains were cultured at 25 °C on nematode growth media agar (NGM) plates containing vehicle (0.1% DMSO), NMN (1 mM in 0.1% DMSO), or #11a (1 mM in 0.1% DMSO) seeded with Escherichia coli strain OP50. Strains were provided by the Caenorhabditis Genetics Center (University of Minnesota). Worm lifespan tests were performed as published.^[^ ^53^ ^]^ Briefly, 30–40 animals were used per condition in each replication. The larvae of each group were raised on NGM plates without any treatment, and were transferred at young adult age to NGM plates with different supplies (vehicle, NMN, or #11a), then scored and transferred every other day.

### The Determination of NAD^+^ Level in *C. Elegans*

For NAD^+^ detection in worm samples, a commercial kit was used for NAD^+^ detection according to the manufacturer's instructions. Briefly, about 100 worms were freshly collected per reaction, quickly washed with M9 buffer three times. Homogenized samples in a 1.5 ml Eppendorf tube with either 100 µl NAD^+^ extraction buffer for NAD^+^ determination or 100 µl NADH extraction buffer for NADH determination. Heated extracts at 60 °C for 5 min, and then added 20 µl Assay Buffer and 100 µl of the opposite extraction buffer to neutralize the extracts. Briefly vortexed and spinned the samples down at 14000 g for 5 min. Prepared 5000 µl 1 µM NAD^+^ Premix by mixing 5 µl 1 mM Standard and 4995 µl distilled water, and diluted in a gradient manner. Added 50 µl Working Reagent per well quickly. Tapped plate to mix. Read fluorescence at λ_ex/em_ = 530/585 nm for time “zero”(F_0_) and F_10_ after a 10‐min incubation at room temperature. Protect plate from light during this incubation. The NAD^+^ content was calculated by establishing a standard curve.

### Real‐Time PCR in *C. Elegans*

Total RNA was isolated from N2 and *cebp‐2* transgenic worms using TRIZOL‐based method. cDNA was synthesized using random primers according to the manufacturer. Real‐time PCR was performed using SYBR green RT‐PCR reagents. The cdc‐42 is used as an internal control gene. Reactions were performed in a QuantStudio three Real‐Time detector (Applied Biosystems). Expression levels of *cebp‐2, lgmn‐1*, and NAD^+^ metabolic enzymes‐encoding genes were normalized to the expression of cdc‐42 using the Ct values.

### Quantification and Statistical Analysis

The statistical analyses were performed with GraphPad Prism 8.0. software. Unless indicated, statistical significance was calculated by unpaired, two‐tailed Student's t‐tests, ordinary ANOVA test for three or more groups (Bonferroni's multiple comparisons test), and Mann‐Whitney U‐tests (when a Gaussian distribution was not assumed). For the Kaplan‐Meier analysis, a log‐rank test was performed. Statistical significance was defined as P < 0.05.

## Conflict of Interest

The authors declare no conflict of interest.

## Author Contributions

K.Y. conceived the project, designed the experiments, analyzed the data, and wrote the manuscript. B.L. designed and performed most of the experiments, analyzed the data, and wrote the manuscript. Z.X. performed the experiments on the *C. elegans*. M.W. performed the cell culture and plasmid construction. S.N. and Z.Q. conducted primary neuronal culture assays. X.M. assisted with data analysis and interpretation. X.L. and S.S.K. performed cognitive behavioral tests and survival curves. All the authors approved the manuscript.

## Supporting information



Supporting Information

## Data Availability

The data that support the findings of this study are available in the supplementary material of this article.
