# Supplementary material for: Neuronal C/EBPβ Shortens the Lifespan via Inactivating NAMPT
Source: Adv Sci (Weinh). 2025 Apr 30;12(21):2414871. doi: 10.1002/advs.202414871 (PMC12140299; doi:10.1002/advs.202414871)
Supplement: Supplementary file 1 — Supporting Information [file ADVS-12-2414871-s001.docx]

Supporting Information

**Neuronal C/EBPβ Shortens the Lifespan via Inactivating NAMPT**

Bowei Li, Zhongyun Xie, Mengmeng Wang, Shuke Nie, Zhengjiang Qian, Xin Meng, Xia Liu, Seong Su Kang, Keqiang Ye.

**This file includes:**

Supplementary Figure S1-S10

Supplementary Tables S1-S4

**Supplementary Figure S1** Analysis of Cebpb and Lgmn genes expression in the brain. A,B) Cebpb and Lgmn expression levels in human brain cells from previously published sequencing data (The Human Protein Atlas).

**Supplementary Figure S2** Characterization of Thy 1-C/EBPβ transgenic mice. A) Schematic diagram of the targeting strategy and allele of Thy 1-C/EBPβ transgenic mice. B) Genotyping of Thy 1-C/EBPβ transgenic mice. C) Western blot verification of human C/EBPβ expression in Thy 1-C/EBPβ Tg mice brain (n = 3). D) Quantification of protein levels in C). E) AEP activity in the hippocampus from WT, Thy 1-C/EBPβ Tg/Tg and Thy 1-C/EBPβ Tg/Tg/AEP^-/-^ mice (n = 3). F) Imaging of WT, Thy 1-C/EBPβ Tg/Tg and Thy 1-C/EBPβ Tg/Tg/AEP^-/-^ mice by using IVIS 100 after injection with LE28 (n = 3). G) Quantification of relative fluorescence intensity in F). All data are presented as the mean ± SEM from 3 to 6 independent experiments. *, P < 0.05; **, P < 0.01; ***, P < 0.001; ****, P < 0.0001.

**Supplementary Figure S3** Antibody specifically recognized the NAMPT (137-491) fragment. A) Sequence alignment of NAMPT protein from indicated species. B) HEK293 cells were transfected with NAMPT FL-GFP or NAMPT 137-491-GFP plasmids, respectively. Western blot showed the specificity of anti-NAMPT C137 antibody. Anti-NAMPT C137 antibody only recognized NAMPT (a.a.137-491) fragment but not full length of NAMPT. C) IF validated the specificity of anti-NAMPT C137 antibody. IF was performed on mouse brain tissues using anti-NAMPT C137 antibody with control peptide or NAMPT peptide antigen (a.a.137-491) for blocking. NAMPT (a.a.137-491) peptide completely blocked the positive signals of anti-NAMPT C137 antibody. Scale bars, 50 μm. D) Quantification of protein levels of AEP, NAMPT C137, EEA1 and LAMP1 in Figure 5E. All data are presented as the mean ± SEM from 3 to 6 independent experiments. *, P < 0.05; **, P < 0.01; ***, P < 0.001; ****, P < 0.0001.

**Supplementary Figure S4** Knockdown of AEP restored NAD^+^ levels and attenuates age-related dysfunction in aged mice. A) Schematic illustrates aged AEP^+/+^ and AEP^+/-^ mice used for analyses. B) Western blot analysis of AEP and NAMPT C137 protein levels in the hippocampus from AEP^+/+^ and AEP^+/-^ mice (n = 3). C) Quantification of protein levels in B). D) IF staining of NeuN, AEP and NAMPT C137 in the indicated group (n = 3). Scale bars, 500 μm, top, 50 μm, bottom. E) Quantification of relative fluorescence intensity in D). F) NAD^+^ levels in the hippocampus from AEP^+/+^ and AEP^+/-^ mice (n = 5). G) Representative traces in the open field test (n = 8). H) Total travelled distance and velocity in G). I) Object recognition memory was assessed by novel object recognition (n = 8). J) Spatial working memory was assessed using the Y maze (n = 8). All data are presented as the mean ± SEM from 3 to 6 independent experiments. *, P < 0.05; **, P < 0.01; ***, P < 0.001; ****, P < 0.0001.

**Supplementary Figure S5** Knockdown of C/EBPβ restored NAD^+^ levels and attenuates age-related dysfunction in aged mice. A) Schematic illustrates aged C/EBPβ^+/+^ and C/EBPβ^+/-^ mice used for analyses. B) Western blot analysis of C/EBPβ, AEP and NAMPT C137 protein levels in the hippocampus from C/EBPβ^+/+^ and C/EBPβ^+/-^ mice (n = 3). C) Quantification of protein levels in B). D) IF staining of NeuN, C/EBPβ, AEP, NAMPT C137 and SIRT1 in the indicated group (n = 3). Scale bars, 500 μm, top, 50 μm, bottom. E) Quantification of fluorescence intensity in D). F) NAD^+^ levels in the hippocampus from C/EBPβ^+/+^ and C/EBPβ^+/-^ mice (n = 5). G) Representative traces in the open field test (n = 8). H) Total travelled distance and velocity in G). I) Object recognition memory was assessed by novel object recognition (n = 8). J) Spatial working memory was assessed using the Y maze (n = 8). All data are presented as the mean ± SEM from 3 to 6 independent experiments. *, P < 0.05; **, P < 0.01; ***, P < 0.001; ****, P < 0.0001.

**Supplementary Figure S6** Blockade of NAMPT cleavage by AEP rescues the age-related dysfunction caused by neuronal C/EBPβ overexpression in mice. A) Quantification of protein levels in Figure 6D. B) Separately graphed (left) male and (right) female total FI scores during lifespan starting at the 1st month in the indicated group. Each dot is the total score of one animal at specific age as indicated. n = all animals alive at each measurement time. C) Time to traverse and number of footslips in beam walking test of Thy 1-C/EBPβ Tg/Tg mice injected with rAAV-hsyn-control, rAAV-hsyn-NAMPT and rAAV-hsyn-NAMPT N136A (n = 8). D) Duration and distance run until exhaustion in high intensity treadmill test (n = 8). All data are presented as the mean ± SEM from 3 to 6 independent experiments. *, P < 0.05; **, P < 0.01; ***, P < 0.001; ****, P < 0.0001.

**Supplementary Figure S7** #11a, NR, or NMN prevents neuronal senescence caused in by C/EBPβ overexpression. LV-C/EBPβ were infected primary neurons at DIV 10 and then were treated with vehicle, #11a, NR, or NMN up to DIV 13. A) Western blot analysis of C/EBPβ, AEP and NAMPT C137 protein levels in primary neurons. B) Quantification of protein levels in A). C) Representative images of SA-β-Gal staining in the indicated group (n = 3). Scale bars, 100 μm. D) Quantification of SA-β-Gal^+^ cells in C). E) NAD^+^ levels in the indicated group (n = 3). F) Representative images of the ratio of aggregates (red)/monomers (green) of JC-1 staining in the indicated group (n = 3). Scale bars, 50 μm. G) Quantification of the ratio of red/green fluorescence in F). H) qPCR analysis of senescence-associated inflammatory cytokine genes in the indicated group (n = 3). All data are presented as the mean ± SEM from 3 to 6 independent experiments. *, P < 0.05; **, P < 0.01; ***, P < 0.001; ****, P < 0.0001.

**Supplementary Figure S8** #11a, NR, or NMN prevents neuronal senescence in Thy1-C/EBPβ Tg/Tg mice. Primary neurons were isolated from WT and Thy 1-C/EBPβ Tg/Tg mice and were monitored at DIV 13. A) Western blot analysis of C/EBPβ, AEP and NAMPT C137 protein levels in primary neurons. B) Quantification of protein levels in A). C) NAD^+^ levels in the indicated group (n = 3). D) Representative images of SA-β-Gal staining in the indicated group (n = 3). Scale bars, 100 μm. E) Quantification of SA-β-Gal^+^ cells in D). F) qPCR analysis of senescence-associated inflammatory cytokine genes in the indicated group (n = 3). G) Representative images of the ratio of aggregates (red)/monomers (green) of JC-1 staining in the indicated group (n = 3). Scale bars, 50 μm. H) Quantification of the ratio of red/green fluorescence in G). Primary neurons isolated from WT and Thy 1-C/EBPβ Tg/Tg mice were treated with vehicle, #11a, NR, or NMN at DIV 10 and were monitored at DIV 13. I) Western blot analysis of C/EBPβ, AEP and NAMPT C137 protein levels in primary neurons. J) Quantification of protein levels in I). K) NAD^+^ levels in the indicated group (n = 3). L) Representative images of SA-β-Gal staining in the indicated group (n = 3). Scale bars, 100 μm. M) Quantification of SA-β-Gal^+^ cells in D). N) qPCR analysis of senescence-associated inflammatory cytokine genes in the indicated group (n = 3). O) Representative images of the ratio of aggregates (red)/monomers (green) of JC-1 staining in the indicated group (n = 3). Scale bars, 50 μm. P) Quantification of the ratio of red/green fluorescence in O). All data are presented as the mean ± SEM from 3 to 6 independent experiments. *, P < 0.05; **, P < 0.01; ***, P < 0.001; ****, P < 0.0001.

**Supplementary Figure S9** Major enzymes involved in NAD^+^ synthesis are dramatically decreased in *unc-119::cebp-2* lines. A) Relative mRNA levels of *cebp-2*(C/EBPβ), *lgmn-1*(AEP) and enzymes involved in NAD^+^ synthesis in wild type (N2) or *unc-119::cebp-2* adult worms were measured using real-time PCR (n = 3). All data are presented as the mean ± SEM from 3 to 6 independent experiments. *, P < 0.05; **, P < 0.01; ***, P < 0.001; ****, P < 0.0001.

**Supplementary Figure S10** #11a inhibits AEP activity and rescues the age-related dysfunction caused by neuronal C/EBPβ overexpression in mice. A) Imaging of Thy 1-C/EBPβ Tg/Tg mice treated with NMN or #11a by using IVIS 100 after injection with LE28 (n = 3). B) Quantification of relative fluorescence intensity in A). C) AEP activity in the hippocampus from Thy 1-C/EBPβ Tg/Tg mice treated with NMN or #11a (n = 3). D) Quantification of protein levels in Figure 7J. E,F) Individually graphed frailty phenotypes that significantly increase with aging, comparing Thy 1-C/EBPβ Tg/Tg mice treated with vehicle, NMN or #11a for male and female. The number of animals assessed at each time point are shown as row beneath each graph. G) Separately graphed (left) male and (right) female total FI scores during lifespan starting at the 1st month. Each dot is the total score of one animal at specific age as indicated. n = all animals alive at each measurement time. H) Time to traverse and number of footslips in beam walking test of Thy 1-C/EBPβ Tg/Tg mice treated with vehicle, NMN or #11a (n = 8). I) Duration and distance run until exhaustion in high intensity treadmill test (n = 8). All data are presented as the mean ± SEM from 3 to 6 independent experiments. *, P < 0.05; **, P < 0.01; ***, P < 0.001; ****, P < 0.0001.

**Supplementary Table S1 Clinical assessment of deficits in mice to create a frailty index.**

| Parameter | Clinical assessment of deficit | Scoring |
| --- | --- | --- |
| Body condition | Place mouse on flat surface, hold tail base and manually assess the flesh/fat that covers the sacroiliac region (back and pubic bones). | 0 = bones palpable, not prominent  0.5 = bones prominent or barely felt  1 = bones very prominent or not felt due to obesity |
| Alopecia | Gently restrain the animal and inspect it for signs of fur loss. | 0 = normal fur density  0.5 = < 25% fur loss  1 = >25% fur loss |
| Coat condition | Inspect the animal for signs of poor grooming. | 0 = smooth, sleek, shiny coat  0.5 = coat is slightly ruffled  1 = unkempt and un-groomed, matted appearance |
| Dermatitis | Document skin lesions. | 0 = absent  0.5 = focal lesions (e.g. neck, flanks, under chin)  1 = widespread or multifocal lesions |
| Eye discharge/swelling | Visual inspection of the mouse to detect ocular discharge and swelling of the eyes. | 0 = normal  0.5 = slight swelling and/or secretions  1 = obvious bulging and/or secretions |
| Forelimb grip  Strength | Hold the mouse. Allow it to grip the bars on the cage lid. Lift animal by the base of the tail to assess grip strength. | 0 = sustained grip  0.5 = reduction in grip strength  1 = no grip strength, no resistance |
| Loss of fur colour | Note any change in fur colour from black to grey or brown. | 0 = normal colour  0.5 = focal grey/brown changes  1 = grey/brown fur throughout body |
| Gait disorders | Observe the freely moving animal to detect abnormalities such as hopping, wobbling, circling, wide stance and weakness. | 0 = no abnormality  0.5 = abnormal gait but animal can still walk  1 = marked abnormality, impairs ability to move |
| Hearing loss | Test startle reflex. Hold a clicker ~10 cm from mouse, sound it 3 times and record responses. | 0 = always reacts (3/3 times)  0.5 = reacts 1/3 or 2/3 times  1 = unresponsive (0/3 times) |
| Kyphosis | Inspect the mouse for curvature of the spine or hunched posture. Run your fingers down both sides of the spine to detect abnormalities. | 0 = absent  0.5 = mild curvature  1 = clear evidence of hunched posture |
| Piloerection | Observe the animal and look for signs of piloerection, in particular on the back of the neck. | 0 = no piloerection  0.5 = involves fur at base of neck only 1 = widespread piloerection |
| Vestibular disturbance | Hold the base of the tail and lower mouse towards a flat surface. Inspect for head tilt, spinning, circling, head tuck or trunk curling. | 0 = absent  0.5 = mild head tilt and/or slight spin when lowered  1 = severe disequilibrium |
| Breathing rate/depth | Observe the animal. Note the rate and depth of breathing as well as any gasping behaviour. | 0 = normal  0.5 = modest change in breathing rate and/or depth  1 = marked changes in rate/depth, gasping |

**Supplementary Table S2 Mouse frailty assessment form****.**

**Date:** ___________________

**Mouse #:** _______________ **Date of Birth:** ______________ **Sex:** F M

**Rating:** 0 = absent 0.5 = mild 1 = severe

_ **Parameter NOTES:**

_ Body condition **0 0.5 1 ___________________________________**

_ Alopecia **0 0.5 1 ___________________________________**

_ Coat condition **0 0.5 1 ___________________________________**

_ Dermatitis **0 0.5 1 ___________________________________**

_ Eye discharge/swelling **0 0.5 1 ___________________________________**

_ Forelimb grip strength **0 0.5 1 ___________________________________**

_ Loss of fur colour **0 0.5 1 ___________________________________**

_ Gait disorders **0 0.5 1 ___________________________________**

_ Hearing loss **0 0.5 1 ___________________________________**

_ Kyphosis **0 0.5 1 ___________________________________**

_ Piloerection **0 0.5 1 ___________________________________**

_ Vestibular disturbance **0 0.5 1 ___________________________________**

_ Breathing rate/depth **0 0.5 1 ___________________________________**

**Total Score/ Max Score:** *________*

**Supplementary Table S3 Human sample characteristics.**

| Age at death | Sex | Number | Date of death | Date of autopsy | Note |
| --- | --- | --- | --- | --- | --- |
| 12Y | Male | 20181228 | 2018/12/28 | 2018/12/28 | Used in Figure 1 |
| 15Y | Male | 20190102 | 2019/1/1 | 2019/1/2 | Used in Figure 1 |
| 18Y | Male | 20191108 | 2019/11/8 | 2019/11/8 | Used in Figure 1 |
| 34Y | Male | 20191021 | 2019/10/21 | 2019/10/21 | Used in Figure 1 |
| 36Y | Male | 20180117 | 2018/1/17 | 2018/1/18 | Used in Figure 1 |
| 36Y | Male | 20190924 | 2019/9/24 | 2019/9/24 | Used in Figure 1 |
| 55Y | Male | 20190417 | 2019/4/17 | 2019/4/17 | Used in Figure 1 |
| 58Y | Male | 20180311 | 2018/03/11 | 2018/03/11 | Used in Figure 1 |
| 58Y | Male | 20190722 | 2019/7/22 | 2019/7/22 | Used in Figure 1 |
| 68Y | Male | 20190220 | 2019/2/20 | 2019/2/20 | Used in Figure 1 |
| 69Y | Female | 20180310 | 2018/3/10 | 2018/3/10 | Used in Figure 1 |
| 69Y | Male | 20190706 | 2019/7/6 | 2018/7/6 | Used in Figure 1 |

Y: year.

**Supplementary Table S4 Key resources table.**

| Reagent or resource | Source | Identifier |
| --- | --- | --- |
| Antibodies |  |  |
| Alexa Fluor® 647 Anti-NeuN antibody | Abcam | ab190565 |
| C/EBPβ antibody (H-7) | Santa Cruz Biotech | Cat#sc-7962 |
| Legumain (D6S4H) Rabbit mAb | Cell Signaling Technology | Cat#93627 |
| Mouse Legumain/Asparaginyl Endopeptidase Antibody | R&D systems | Cat#AF2058 |
| NAMPT/PBEF Polyclonal antibody | Proteintech | Cat#11776-1-AP |
| NAMPT C137 antibody | Sangon Biotech | N/A |
| SIRT1 antibody (B-7) | Santa Cruz Biotech | Cat#sc-74465 |
| 4 Hydroxy-nonenal | Abcam | Cat#ab46545 |
| p21 Cip1 Antibody | Affinity Biosciences | Cat#AF6290 |
| GAPDH Monoclonal antibody | ProteinTech | Cat#60004 |
| GST Tag Monoclonal antibody | Proteintech | Cat#66001 |
| MYC tag Polyclonal antibody | Proteintech | Cat#16286 |
| Goat anti-rabbit secondary antibody | ABclonal Technology | Cat#AS014 |
| Goat anti-mouse secondary antibody | ABclonal Technology | Cat#AS003 |
| Donkey anti-Mouse IgG (H+L) Highly Cross-Adsorbed Secondary Antibody, Alexa Fluor™ 488 | Thermo Fisher Scientific | Cat#A21202 |
| Donkey anti-Rabbit IgG (H+L) Highly Cross-Adsorbed Secondary Antibody, Alexa Fluor™ 555 | Thermo Fisher Scientific | Cat#A31572 |
| Donkey anti-Goat IgG (H+L) Highly Cross-Adsorbed Secondary Antibody, Alexa Fluor™ Plus 647 | Thermo Fisher Scientific | Cat#A32849TR |
| Donkey anti-Sheep IgG (H+L) Cross-Adsorbed Secondary Antibody, Alexa Fluor™ 647 | Thermo Fisher Scientific | Cat#A21448 |
| Chemicals, peptides, and recombinant proteins |  |  |
| LE28 | Edgington-Mitchell Lab | N/A |
| DAPI | Sigma-Aldrich | D9542 |
| AEP substrate Z-Ala-Ala-Asn-AMC | Bachem | Cat#4033201 |
| Triton X-100 | Sigma-Aldrich | Cat#X100 |
| TRIzol Reagent | Life Technologies | Cat#15596018 |
| Nicotinamide mononucleotide | Bontac Bio-Engineering, China | N/A |
| Polybrene | Sigma-Aldrich | Cat#H9268 |
| DMEM Medium | Gibco | Cat#11965-092 |
| DMEM/F-12 Medium | Gibco | Cat#11320-033 |
| Endothelial Cell Culture Medium | CTCC | Cat#CTCC-002-031 |
| Penicillin-streptomycin | Gibco | Cat#15140 |
| Dithiothreitol (DTT) | Roche | Cat#10708984001 |
| Normal Donkey Serum | Jackson Immuno Research | Cat#017-000-121 |
| Bovine Serum Albumin (BSA) | Sangon Biotech | Cat#A600332-0100 |
| EDTA, 0.5M | Thermo Fisher Scientific | Cat#AM9261 |
| Lipofectamine 3000 | Thermo Fisher Scientific | Cat#L3000008 |
| Critical commercial assays |  |  |
| NAD^+^/NADH assay Kit | Bioassay Systems | Cat#EFND-100 |
| JC-1 Mitochondrial Membrane Potential Detection Kit | Beyotime | Cat#C2006 |
| Rapid Site-Specific Mutagenesis Kit | TIANGEN | Cat#KM101 |
| Plasmid Extraction Mini Kit | TIANGEN | Cat#DP103 |
| PrimeScript™ RT reagent Kit with gDNA Eraser | Takara | RR047Q |
| UNICON® qPCR SYBR Green Master Mix | Yeasen | #11184ES08 |
| Agarose gel DNA Recovery Kit | TIANGEN | Cat#DP209 |
| Experimental Models: Cell Lines |  |  |
| HEK293T | ATCC | Cat#CRL-3216 |
| Experimental models: Organisms/strains |  |  |
| Mouse: C57/BL6J | Jackson Laboratory | 000664 |
| Mouse: Thy 1-C/EBPβ | Cyagen | N/A |
| Mouse: AEP^+/-^ | Ye Lab | N/A |
| Mouse: C/EBPβ^+/-^ | Ye Lab | N/A |
| Oligonucleotides |  |  |
| Il1b forward 5’ GAAATGCCACCTTTTGACAGTG | Sangon Biotech | N/A |
| Il1b reverse 5’ TGGATGCTCTCATCAGGACAG | Sangon Biotech | N/A |
| Tnfa forward 5’ TGCCTATGTCTCAGCCTCTTC | Sangon Biotech | N/A |
| Tnfa reverse 5’ GAGGCCATTTGGGAACTTCT | Sangon Biotech | N/A |
| Ifng forward 5’ GCCACGGCACAGTCATTGA | Sangon Biotech | N/A |
| Ifng reverse 5’ TGCTGATGGCCTGATTGTCTT | Sangon Biotech | N/A |
| Gapdh forward 5’ ACTCCACTCACGGCAAATTCAACG | Sangon Biotech | N/A |
| Gapdh forward 5’ AAGACACCAGTAGACTCCACGACA | Sangon Biotech | N/A |
| Recombinant DNA |  |  |
| Plasmid myc-AEP | Ye Lab | N/A |
| Plasmid myc-AEP C189S | Ye Lab | N/A |
| Plasmid GST vector | Sangon Biotech | N/A |
| Plasmid GST-NAMPT | This study | N/A |
| Plasmid GST-NAMPT N136A | This study | N/A |
| Plasmid GST-NAMPT 1-136 | This study | N/A |
| Plasmid GST-NAMPT 137-491 | This study | N/A |
| Software and Algorithms |  |  |
| GraphPad Prism v8 | GraphPad Software | https://www.graphpad.com/scientificsoftware/  prism/ |
| Fiji | ImageJ | http://imagej.net/Fiji/Downloads |
| ZEN 2012 | Zeiss | https://www.zeiss.com/microscopy/en/home.html |
